# Supplementary material for: Tsc1 represses parvalbumin expression and fast-spiking properties in somatostatin lineage cortical interneurons
Source: Nat Commun. 2019 Nov 1;10:4994. doi: 10.1038/s41467-019-12962-4 (PMC6825152; doi:10.1038/s41467-019-12962-4)
Supplement: Supplementary file 1 — Supplementary Information [file 41467_2019_12962_MOESM1_ESM.pdf]

Tsc1 represses parvalbumin expression and fast spiking properties in somatostatin lineage cortical interneurons

Malik et al.

Supplementary Figures

Supplementary Fig. 1

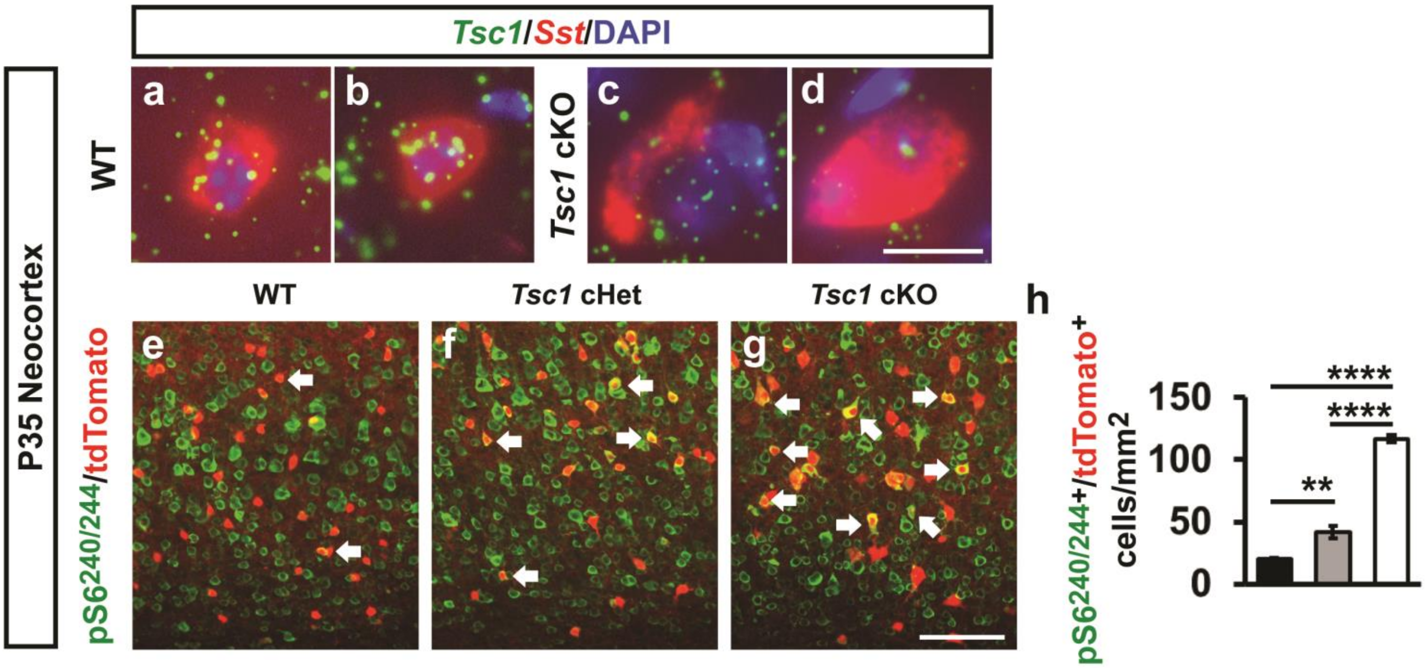

Validation of *Tsc1* loss in *SST-Cre*-lineage CINs and MTOR activity

(a-d) *Tsc1* (green) and *Sst* (red) fluorescent *in situ* hybridization from WT **(a-b)** and *Tsc1* cKO **(c-d)** P35 neocortex. **(e-g)** *SST*-*Cre*-lineage CINs (tdTomato+) co-labeled for the MTOR target, phosphorylated ribosomal subunit S6 at serines 240 and 244 (green). **(h)** Quantification of dual labelled pS6/tdTomato cell density in the neocortex: One-way ANOVA ($F_{2, 6}$ = 228.6, P < 0.0001; cKO veh vs. all groups, p < 0.0001; WT vs. cHet, p = 0.0093). Data are expressed as the mean ± SEM, n = 3, all groups. ** p < 0.01, **** p < 0.0001. Scale bars in **(d)** = 20µm and **(g)** = 100µm. Source data are provided as a Source Data file.

Supplementary Fig. 2

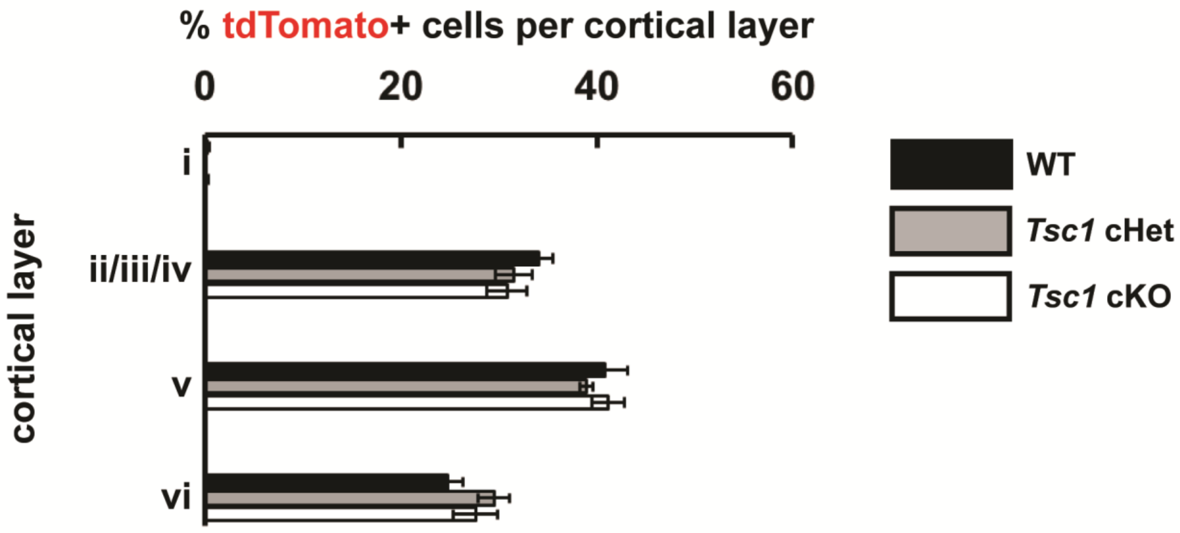

Normal lamination distribution of *SST-Cre; Tsc1* mutants

P35 coronal immunofluorescent tissue sections were assessed for tdTomato+ cells in cortical lamina of the somatosensory cortex. Graph shows the % of tdTomato cells that occupy cortical layers i, ii/iii/iv, v and vi for each genotype: Chi-squared test with Yate's correction found no significant differences, n = 3 mice per genotype; 441 (WT), 429 (cHet) and 494 (cKO) total cells counted. Data are expressed as the mean ± SEM. Source data are provided as a Source Data file.

Supplementary Fig. 3

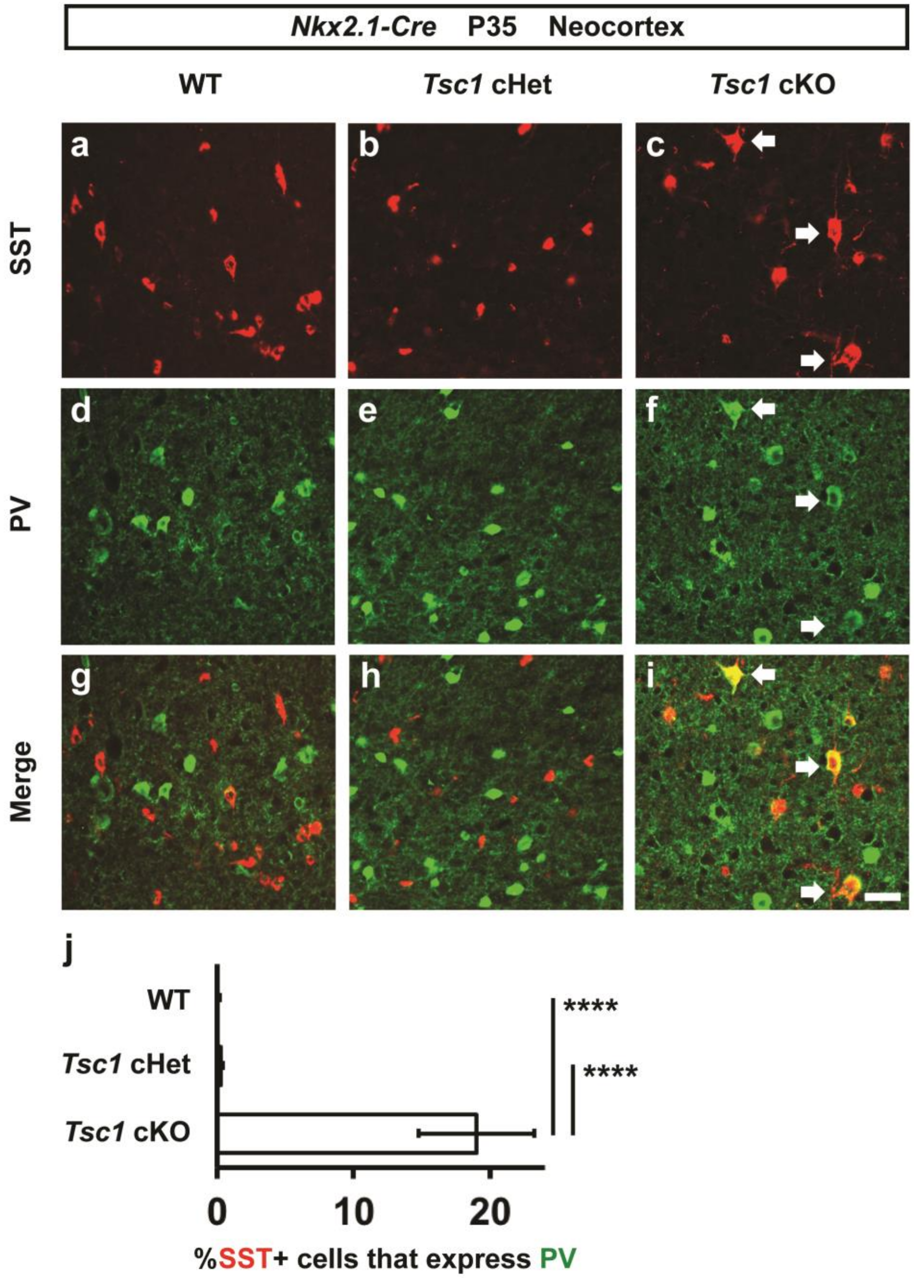

Co-expression of SST and PV in the somatosensory cortex of *Nkx2.1-Cre; Tsc1* cKOs

P35 coronal immunofluorescent tissue sections were assessed for somatostatin (SST) **(a-c)** and parvalbumin (PV) **(d-f)** in the somatosensory cortex. **(g-i)** SST and PV merged images; note that *Tsc1* cKO neocortices had multiple co-labeled cells. Arrows point to co-labeled cells. **(j)** Quantification of the % of SST+ CINs co-labeled for PV: (Chi-squared test with Yate's correction **** $p < 0.0001$, n = 3 mice, all groups; 872 WT cells, 1035 cHet cells and 762 cKO tdTomato+ cells assessed). Data are expressed as the mean ± SEM. Scale bar in **(i)** = 100µm. Source data are provided as a Source Data file.

Supplementary Fig. 4

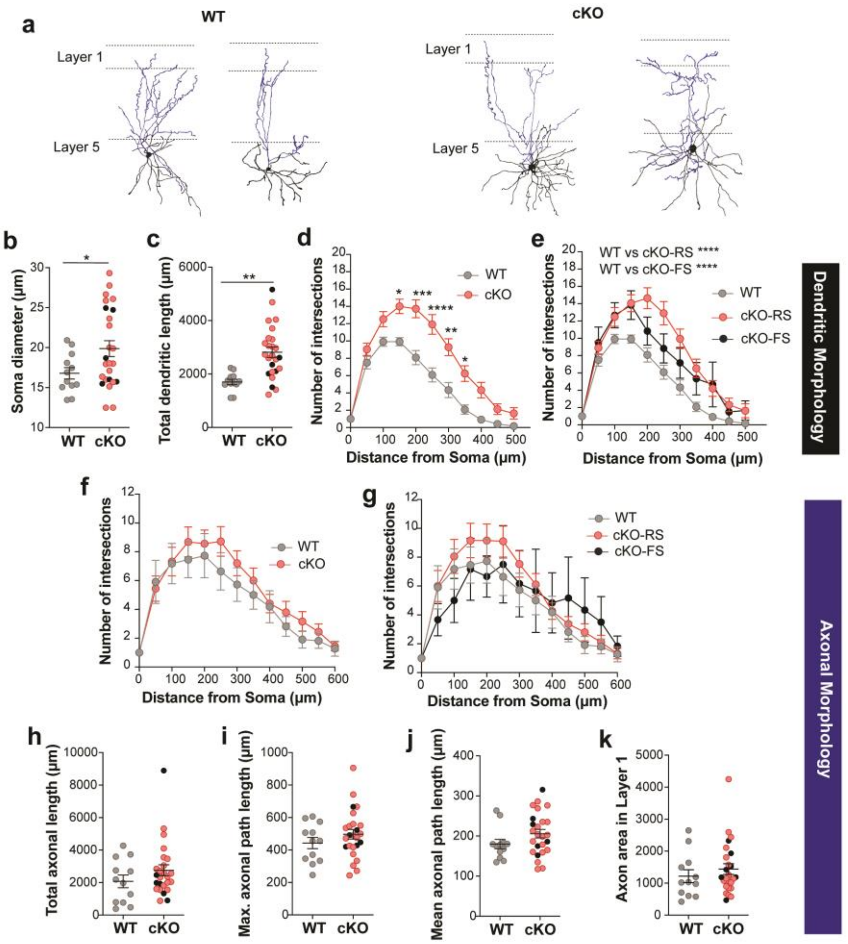

***Tsc1* deletion increases dendritic complexity of *SST-lineage* CINs**

(a) Example tracings of two SST+ CINs from WTs and cKOs. Dendrites and somas are shown in black, axons are shown in blue. **(b)** Increased soma diameter of cKO CINs (red circles, 19 neurons) in comparison with WT CINs (grey circles, 12 neurons). Black filled circles denote cKO CINs (6 neurons) with fast spiking physiology (t_{39} = 2.02, p = 0.03). **(c)** Same as **b** for total dendritic length (t_{39} = 3.28, p = 0.002). **(d)** Number of intersections of dendritic branches with Sholl circles. Note, the cKO CINs had significantly more intersections (Two-way ANOVA, $F_{10, 385}$ = 2.2, P = 0.01). **(e)** Number of intersections of dendritic branches with Sholl circles is compared for WT (grey), cKOs with regular spiking physiology (red) and cKOs with fast spiking physiology (black) (Two way ANOVA, $F_{20, 374}$ = 1.6, P = 0.04; WT vs. cKO RS, p < 0.0001; WT vs. cKO FS, p < 0.0001). **(f)** Same as **d** for axonal branches (Two-way ANOVA, $F_{12, 442}$ = 0.25, P = 0.99). **(g)** Same as **e** for axonal branches (Two-way ANOVA, $F_{24, 429}$ = 0.55, P = 0.9). **(h)** Comparison of total axonal length (t_{35} = 1.2, p = 0.23). **(i)** Comparison of maximum axonal path length (t_{35} = 1.1, p = 0.27). **(j)** Comparison of mean axonal path length (t_{39} = 1.5, p = 0.14). **(h)** Comparison of axon surface area in Layer 1 (t_{35} = 0.82, p = 0.42). Data are presented as mean ± S.E.M. * p < 0.05; ** p < 0.01, *** p < 0.001, **** p < 0.0001. Source data are provided as a Source Data file.

Supplementary Fig. 5

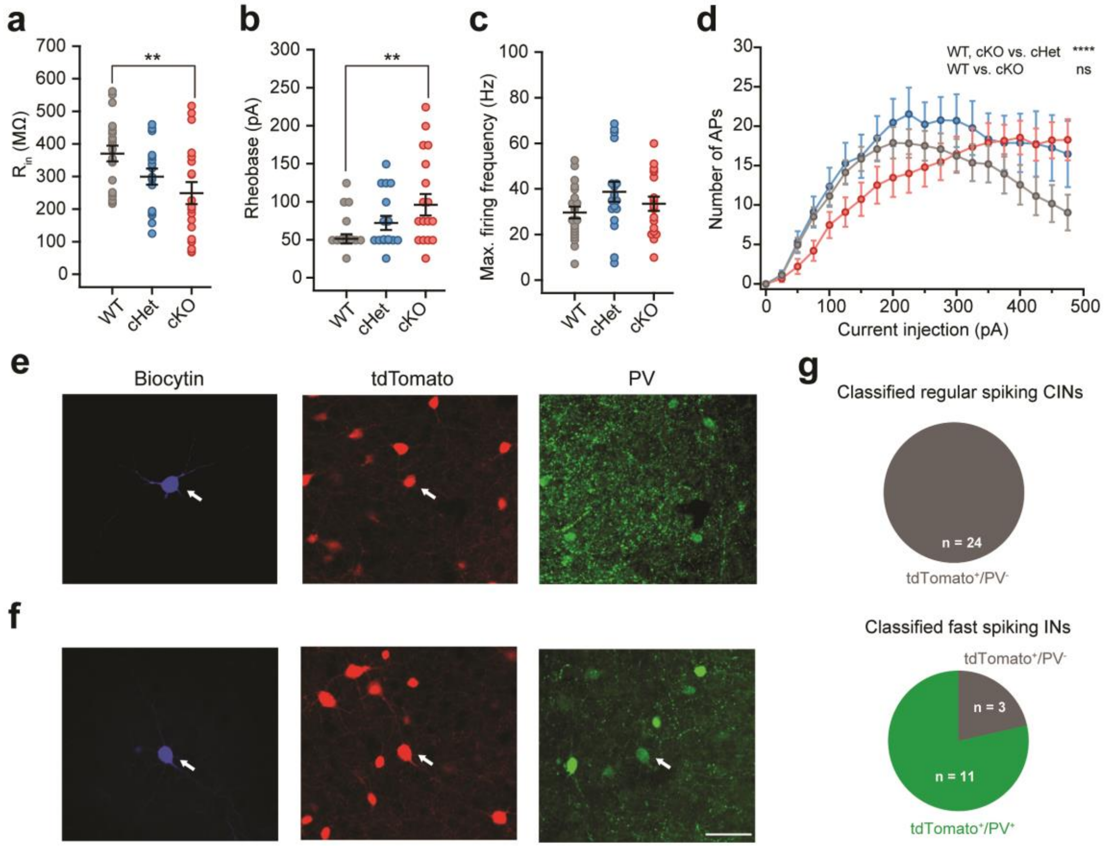

***SST-Cre* lineage CINs with fast-spiking properties express PV and contribute to the increased firing output in cKOs**

(a) Comparison of input resistance (R_{in}): One-way ANOVA ($F_{2,\ 54}$ = 5.02, P = 0.007) **(a)**; rheobase, One-way ANOVA ($F_{2,\ 54}$ = 5, p = 0.007, WT vs cKO, p = 0.007)) **(b);** and maximum firing frequency, One-way ANOVA ($F_{2,\ 54}$ = 1.77, WT vs cKO, p = 0.007) **(c)** of regular-spiking SST-lineage CINs in WT (grey), cHet (blue) and cKO (red) groups. Note the R_{in} of regular-spiking CINs in cKO group is significantly lower and rheobase is higher as compared to WT group (WT, n=21 cells from 4 mice; cHet, n=17 cells from 3 mice; cKO, n=19 cells from 4 mice). **(d)** Number of APs fired in response to steps of depolarizing current injections: Two-way ANOVA ($F_{(2,1079)}$ = 11.1, P < 0.0001; WT and cKO vs. cHet, p < 0.0001; WT vs. cKO, p = 0.97). Data are presented as mean ± S.E.M. **p < 0.01, **** p < 0.0001. **(e)** Layer 5 *SST-lineage* CIN in *Tsc1* cKO was filled with biocytin (grey) and later confirmed to express tdTomato (red) but not PV (green). **(f)** Layer 5 *SST-lineage* CIN in cKO filled with biocytin and later confirmed to express both tdTomato and PV. **(g)** Top: Pie chart showing the proportion of biocytin-filled CINs in cKOs and cHets classified as RS that also expressed PV. Bottom: Pie chart showing the proportion of biocytin-filled CINs in cKOs and cHets classified as FS that also expressed PV. Scale bar in **(f)** = 50µm. Source data are provided as a Source Data file.

Supplementary Fig. 6

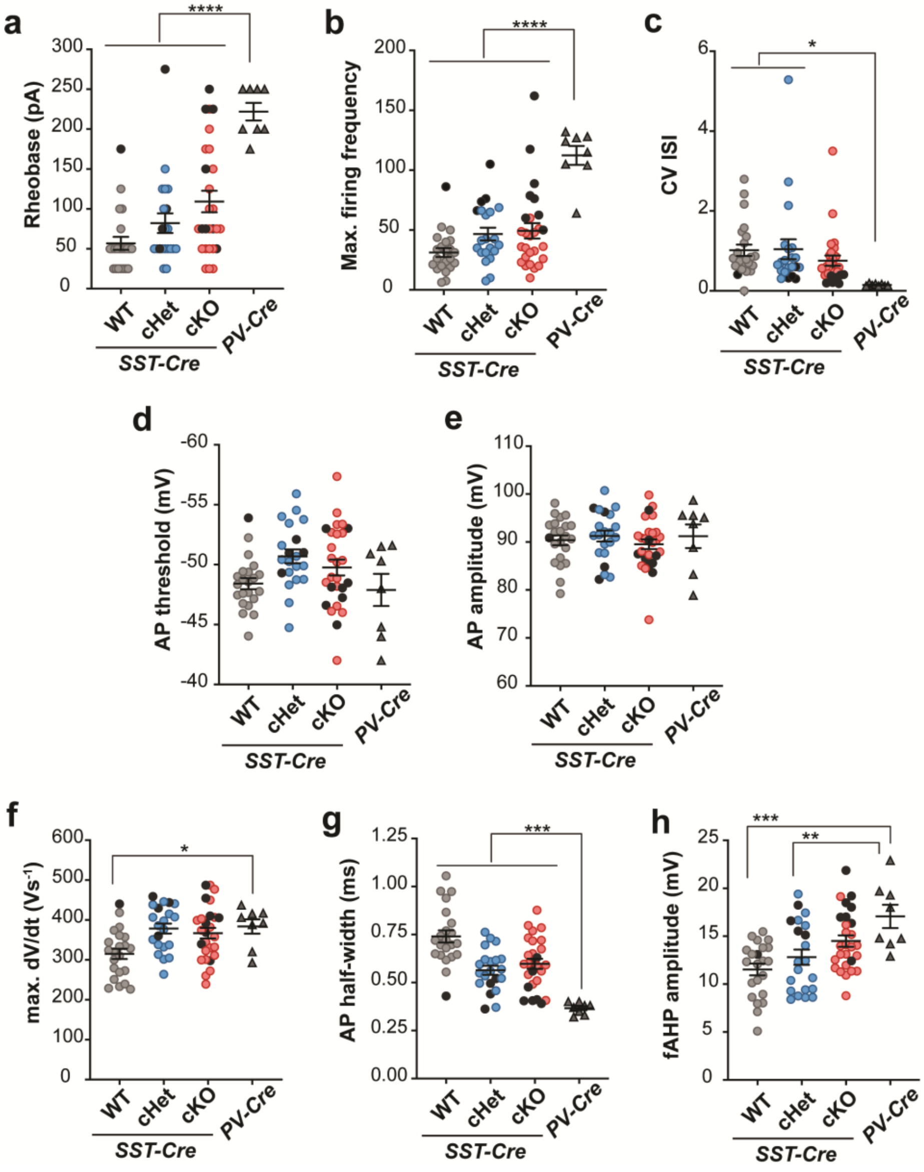

Comparison of firing properties of SST+ CINs and PV+ CINs

(a) Comparison of rheobase of *SST-Cre* WT (grey circles, n = 22 cells from 4 mice), *SST-Cre; Tsc1* cHet (blue circles, n = 21 cells from 3 mice), *SST-Cre; Tsc1* cKO (red circles, n = 27 cells from 4 mice) and *PV-Cre* CINs (grey triangles, n = 8 cells from 1 mouse). *SST-Cre* CINs with fast spiking physiological properties are plotted as black filled circles. ($F_{3, 74}$ = 18.2, P < 0.00001; *SST-Cre* WT vs. *PV-Cre* p <0.0001, *SST-Cre* cHet vs. *PV-Cre* p <0.0001, *SST-Cre* cKO vs. *PV-Cre* p <0.0001), One-way ANOVA with Bonferroni's multiple comparisons test. **(b)** Comparison of maximum firing frequency, ($F_{3, 74}$ = 19.14, P < 0.0001; *SST-Cre* WT vs. *PV-Cre* p < 0.0001, *SST-Cre* cHet vs. *PV-Cre* p < 0.0001, *SST-Cre* cKO vs. *PV-Cre* p < 0.00019), One-way ANOVA with Bonferroni's multiple comparisons test. **(c)** Comparison of coefficient of variance in inter-spike interval (CV ISI), ($F_{3, 74}$ = 2.94, P = 0.03; *SST-Cre* WT vs. *PV-Cre* p = 0.03, *SST-Cre* cHet vs. *PV-Cre* p = 0.025, *SST-Cre* cKO vs. *PV-Cre* p =

0.18), One-way ANOVA with Bonferroni's multiple comparisons test. **(d)** Comparison of action potential (AP) threshold, ($F_{3, 74}$ = 2.9, P = 0.03; *SST-Cre* WT vs. *PV-Cre* p > 0.9, *SST-Cre* cHet vs. *PV-Cre* p = 0.07, *SST-Cre* cKO vs. *PV-Cre* p = 0.35), One-way ANOVA with Bonferroni's multiple comparisons test. **(e)** Comparison of AP amplitude, ($F_{3, 74}$ = 0.48, P = 0.69), One-way ANOVA. **(f)** Comparison of AP maximum dV/dt, ($F_{3, 74}$ = 4.96, P = 0.003; *SST-Cre* WT vs. *PV-Cre* p = 0.026, *SST-Cre* cHet vs. *PV-Cre* p > 0.9, *SST-Cre* cKO vs. *PV-Cre* p > 0.9), One-way ANOVA with Bonferroni's multiple comparisons test. **(g)** Comparison of AP half-width, ($F_{3, 74}$ = 18.6, P < 0.0001; *SST-Cre* WT vs. *PV-Cre* p < 0.0001, *SST-Cre* cHet vs. *PV-Cre* p = 0.002, *SST-Cre* cKO vs. *PV-Cre* p = 0.0001), One-way ANOVA with Bonferroni's multiple comparisons test. **(h)** Comparison of fast afterhyperpolarization (fAHP) amplitude, ($F_{3, 74}$ = 7.14, P = 0.0003; *SST-Cre* WT vs. *PV-Cre* p = 0.0003, *SST-Cre* cHet vs. *PV-Cre* p = 0.006, *SST-Cre* cKO vs. *PV-Cre* p = 0.15), One-way ANOVA with Bonferroni's multiple comparisons test. Data are presented as mean ± S.E.M. * p < 0.05; ** p < 0.01, *** p < 0.001, **** p < 0.0001. Source data are provided as a Source Data file.

Supplementary Fig. 7

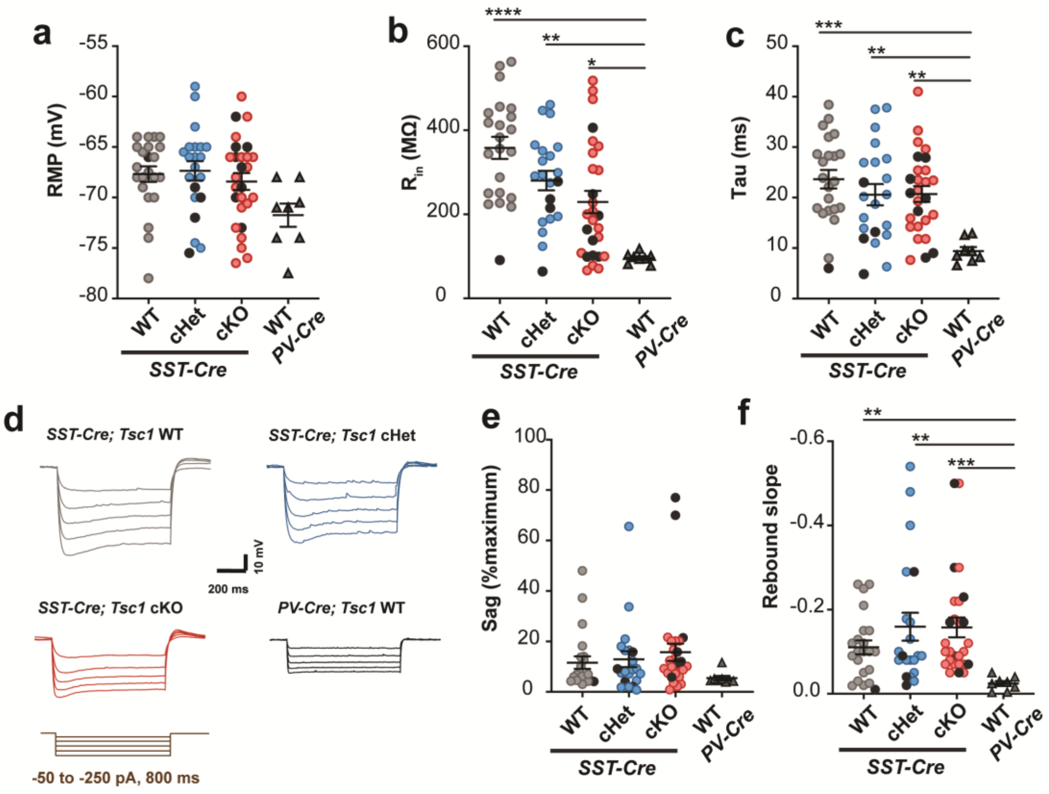

Comparison of subthreshold membrane properties of SST+ CINs and PV+ CINs

(a) Resting membrane potential (RMP) of *SST-Cre* WT (grey circles, n = 22 cells from 4 mice), *SST-Cre; Tsc1* cHet (blue circles, n = 21 cells from 3 mice), *SST-Cre; Tsc1* cKO (red circles, n = 27 cells from 4 mice) and *PV-Cre* CINs (grey triangles, n = 8 cells from 1 mouse) are plotted ($F_{3, 74}$ = 2.46, P = 0.068). *SST-Cre* CINs with fast spiking physiological properties are plotted as black filled circles. **(b)** Comparison of input resistance (R_{in}), ($F_{3, 74}$ = 10.8, P < 0.0001; *SST-Cre* WT vs. *PV-Cre* p <0.0001, *SST-Cre* cHet vs. *PV-Cre* p = 0.001, *SST-Cre* cKO vs. *PV-Cre* p = 0.02), One-way ANOVA with Bonferroni's multiple comparisons test. **(c)** Comparison of membrane time constant (Tau), ($F_{3, 74}$ = 5.7, P = 0.001; *SST-Cre* WT vs. *PV-Cre* p = 0.0003, *SST-Cre* cHet vs. *PV-Cre* p = 0.005, *SST-Cre* cKO vs. *PV-Cre* p = 0.003), One-way ANOVA with Bonferroni's multiple comparisons test. **(d)** Example voltage traces in response to increasing steps of hyperpolarizing current injections (brown) in *SST-Cre* CINs from WT (grey), *Tsc1* cHet (blue) and *Tsc1* cKO mice (red), and *PV-Cre* CINs (black). **(e)** Comparison of maximum sag voltage (%), (Kruskal-Wallis statistic = 7.38, P = 0.06), Kruskal-Wallis ANOVA. **(f)** Comparison of rebound slope, (Kruskal-Wallis statistic = 17.67, P = 0.0005; *SST-Cre* WT vs. *PV-Cre* p = 0.004, *SST-Cre* cHet vs. *PV-Cre* p = 0.001, *SST-Cre* cKO vs. *PV-Cre* p = 0.0001), Kruskal-Wallis ANOVA with Dunn's multiple comparison test. Data are presented as mean ± S.E.M. * p < 0.05, ** p < 0.01, *** p < 0.001, **** p < 0.0001. Source data are provided as a Source Data file.

Supplementary Fig. 8

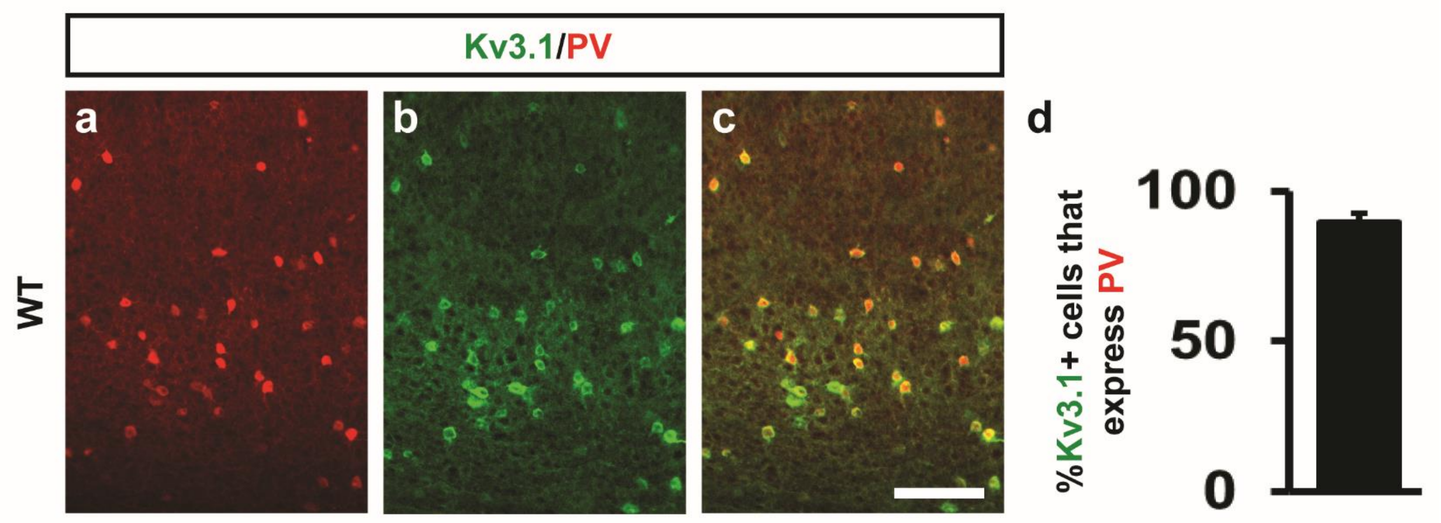

Validation of preferential expression of Kv3.1 in PV+ CINs

(a-c) P35 WT neocortices were co-labeled for parvalbumin (PV) and the potassium channel, Kv3.1. Scale bar in **(c)** = 100µm. **(d)** quantification of the proportion of Kv3.1+ cells that express PV. Data are expressed as the mean ± SEM. n = 3 mice. Source data are provided as a Source Data file.

Supplementary Fig. 9

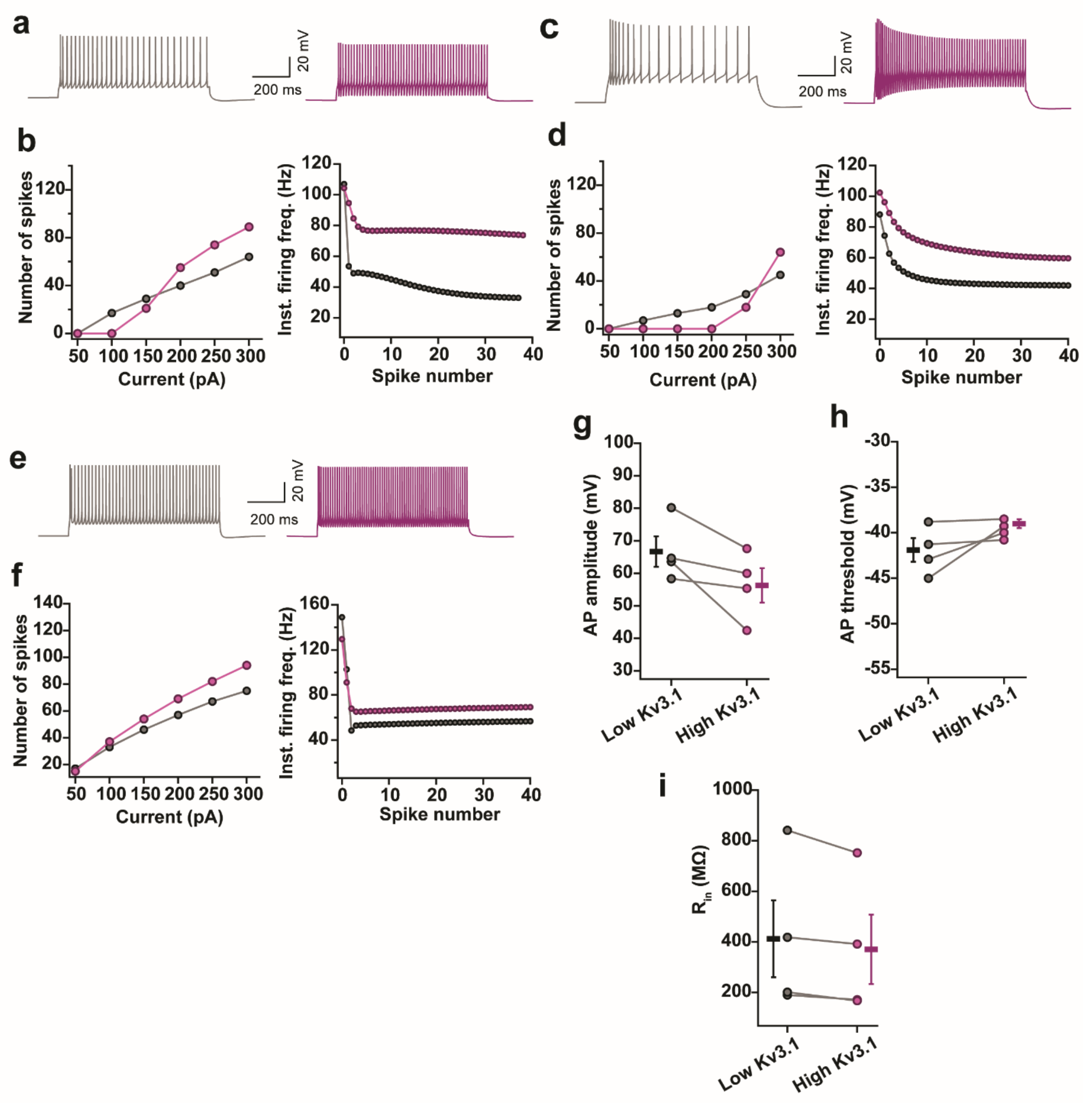

Increased Kv3.1 conductance switches the physiological properties of SST+ CINs from regular spiking to fast spiking

(a) Example firing traces from a morphologically realistic model of SST+ CINs with low expression of Kv3.1 (grey) and high expression of Kv3.1 (purple). **(b)** Firing data from one model is shown. Increasing the conductance of Kv3.1 channel increases the number of APs fired in response to depolarizing current injections (left) and decreases the change in instantaneous firing frequency (right) during a train of APs. **(c–f)** Same as **(a, b)** for two other models of SST+ CINs models. **(g–i)** Increasing the Kv3.1 conductance in four different models of SST+ CINs does not affect the AP amplitude (Paired two-tailed t-test, t_3 = 2.48), AP threshold (Paired two-tailed t-test, t_3 = 1.8) and input resistance (R_{in}) (Paired two-tailed t-test, t_3 = 2.66). Data are presented as mean ± S.E.M.

Supplementary Fig. 10

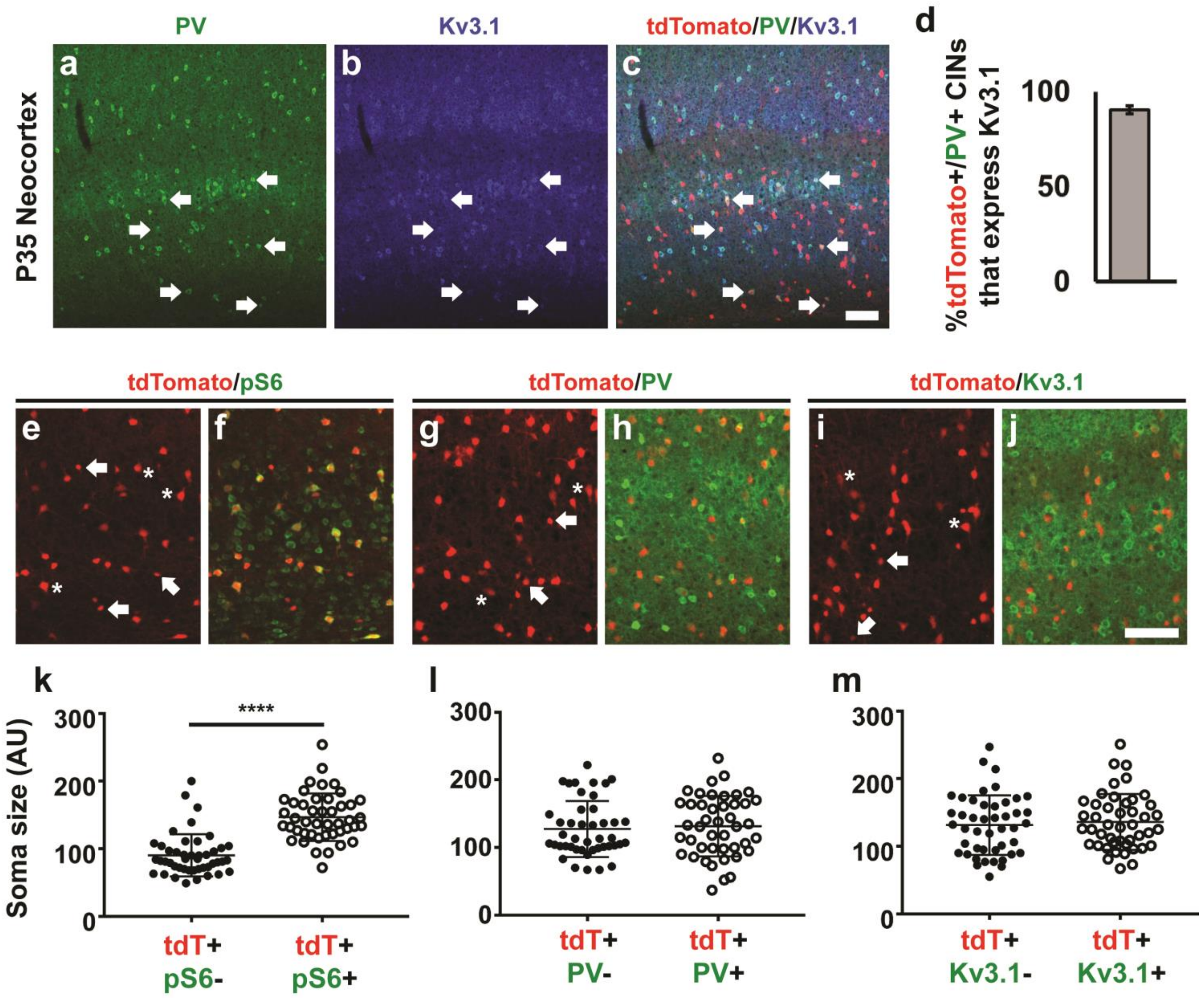

Increased soma size correlates with pS6 but not PV or Kv3.1 expression

(a-c) Immuno-fluorescent images of dual labeled *SST-Cre* lineage (tdTomato+)/PV+ CINs that co-express Kv3.1 in *Tsc1* cKO neocortices. **(d)** Quantification of the % dual tdTomato+/PV+ CINs that express Kv3.1. tdTomato+ CINs co-labeled for pS6 **(e-f)**, PV **(g-h)** or Kv3.1 **(i-j)**. Arrows denote smaller soma CINs; asterisks denote larger soma CINs. **(k-m)** Soma size quantification of CINs that are tdTomato+ and are either negative (-) or positive (+) for the indicated marker: Two-tailed T-test ($t = 8.04$, $df = 88$), **** $p < 0.0001$. $n = 75$ both pS6 groups, $n = 74$ both groups of PV and $n = 75$ both groups of Kv3.1. Data are presented as mean ± S.E.M. **(d)** or ±SD **(k-m)**. Scale bars in **(c)** and **(j)** = 100µm. Source data are provided as a Source Data file.

Supplementary Fig. 11

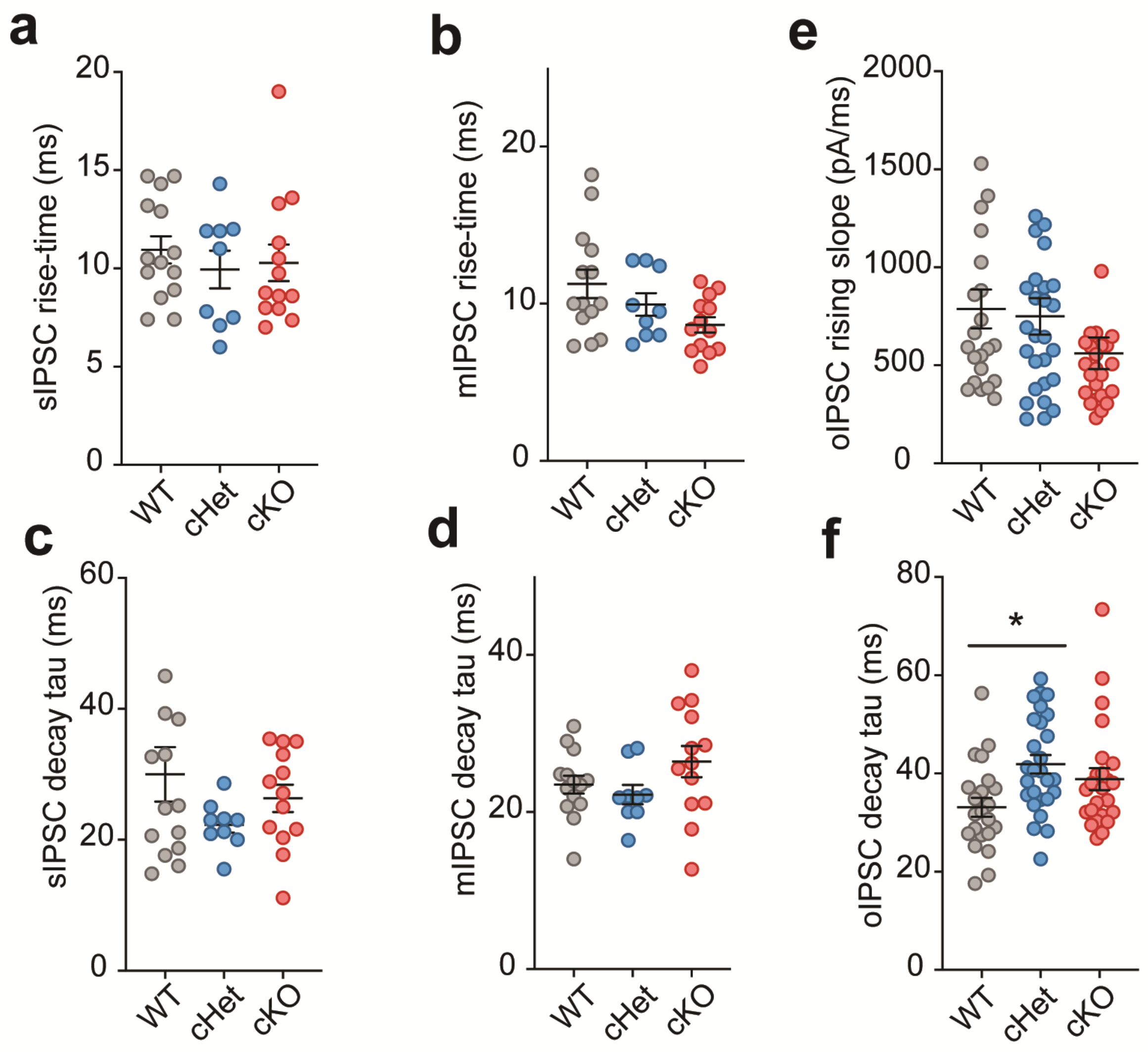

The kinetics of inhibitory currents are not affected by *Tsc1* deletion

(a, b) The rise-time of sIPSCs and mIPSCs in WTs (15 cells from 3 mice), cHets (9 cells from 2 mice) and cKOs (13 cells from 3 mice) is compared (One-way ANOVA; sIPSC rise-time: $F_{2,33} = 1.1$; mIPSC rise-time: $F_{2,33} = 3.1$). **(c, d)** The decay tau of sIPSCs and mIPSCs in WTs, cHets and cKOs is compared (One-way ANOVA; sIPSC decay-time: $F_{2,33} = 1.4$; mIPSC decay-time: $F_{2,33} = 1.8$). **(e, f)** The rising slope (One-way ANOVA; oIPSC rise-time: $F_{2,66} = 1.8$) and decay tau (One-way ANOVA; sIPSC decay-time: $F_{2,66} = 4.4$; WT vs cHet, Tukey's post-hoc test $p = 0.01$) of optogenetically evoked inhibitory currents (oIPSCs) mediated by SST+ CINs in WTs, cHets, cKOs is compared. Data are presented as mean ± S.E.M. * $p < 0.05$. Source data are provided as a Source Data file.

Supplementary Fig. 12

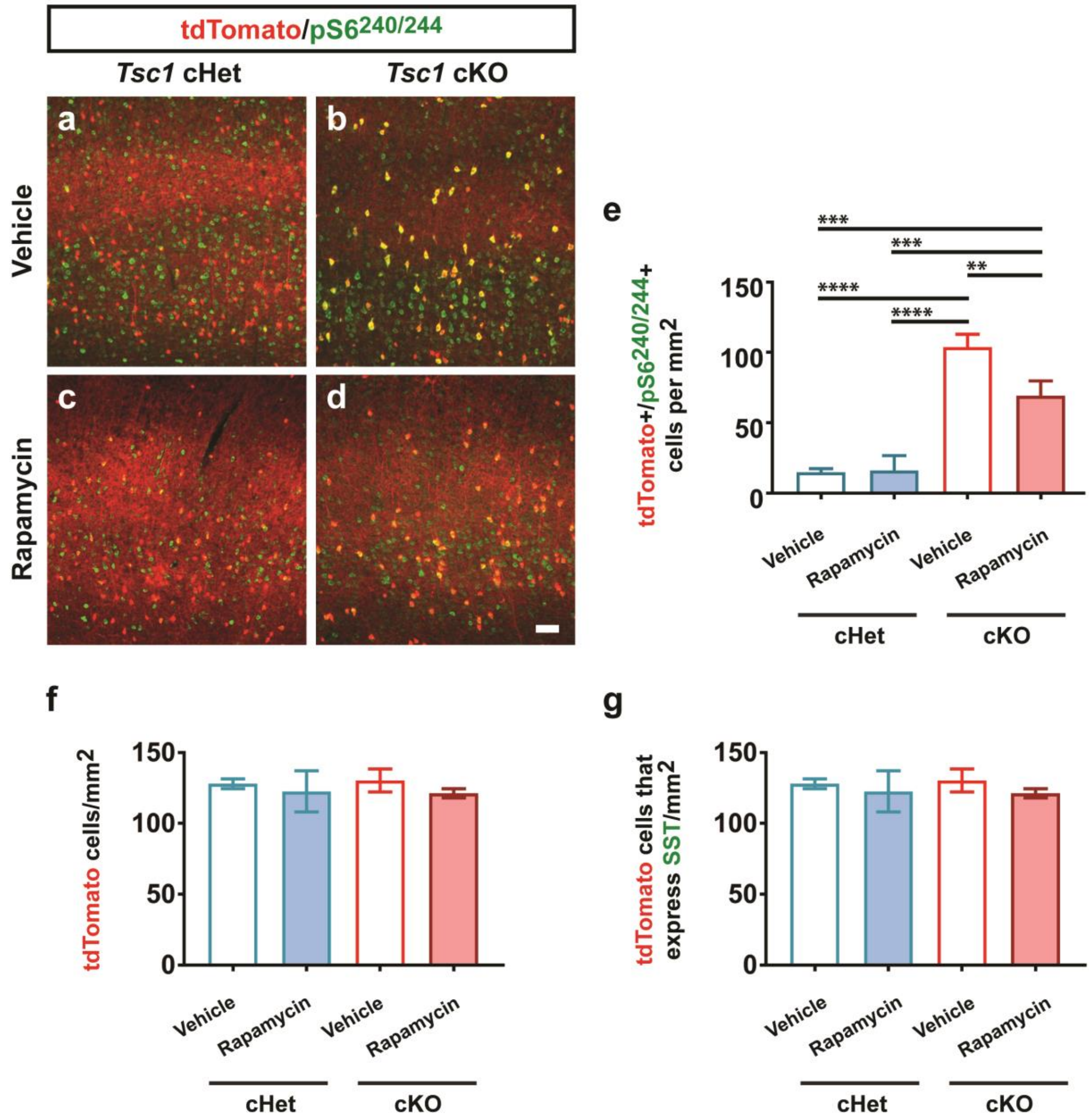

Validation of rapamycin treated mice and additional cell counts

Vehicle treated **(a, b)** or rapamycin treated **(c, d)** *Tsc1; SST-Cre* cHet and cKO neocortices were immuno-fluorescently labeled for the MTOR target, phosphorylated ribosomal subunit $S6^{Ser240/244}$. **(e)** quantification of the cell density of *SST-Cre*-lineage cells (tdTomato+) that co-label for pS6 in the neocortex: One-way ANOVA ($F_{3, 8}$ = 73.16, P < 0.0001; cKO veh vs. cHet veh and cHet rapa p < 0.0001; cKO veh vs. cKO rapa p = 0.0059; cHet veh vs. cKO rapa p = 0.0003; cHet rapa vs. or cKO rapa p = 0.0003). **(f)** quantification of the cell density of tdTomato+ cells: One-way ANOVA ($F_{3, 8}$ = 0.9). **(g)** quantification of the cell density of tdTomato+ cells that co-express SST: One-way ANOVA ($F_{3, 8}$ = 1.8). Data are represented as mean ± SEM. Scale bar in **(d)** = 100µm. n = 3, all groups. * p < 0.05, **** p < 0.0001. Source data are provided as a Source Data file.

Supplementary Fig. 13

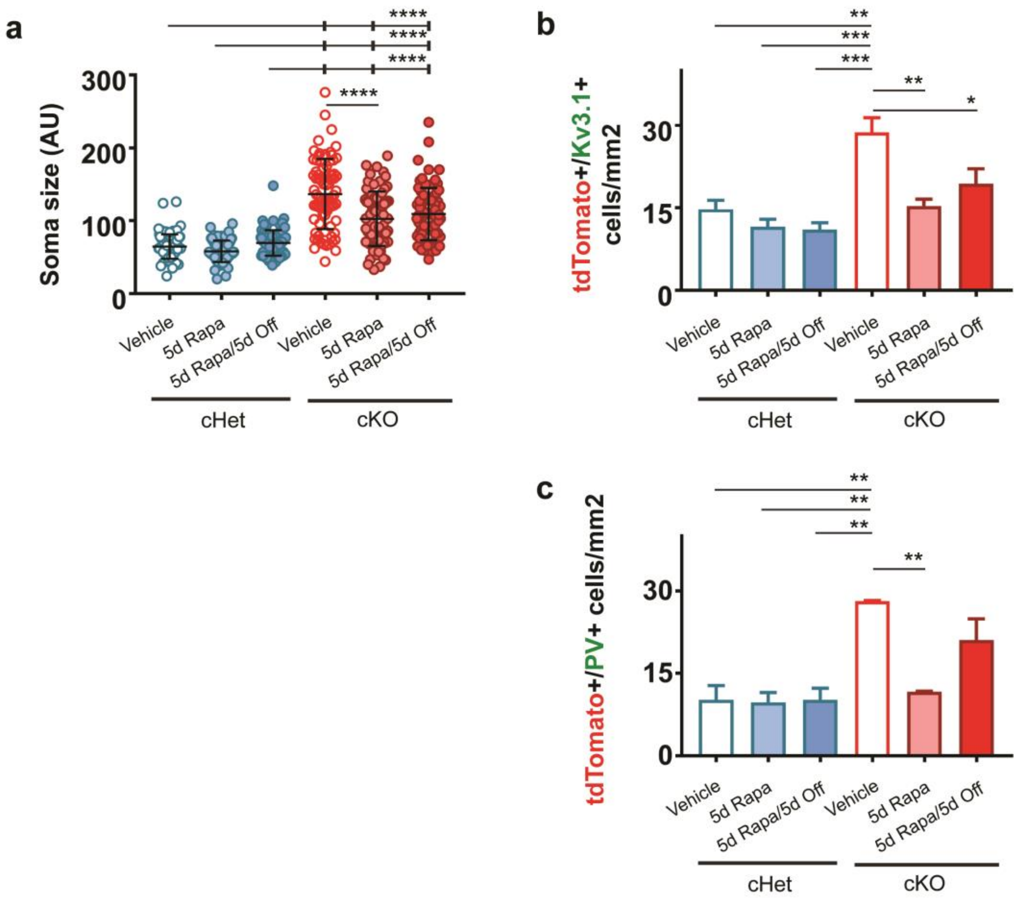

Effects of rapamycin removal on soma size, PV and Kv3.1 expression

8-week old mice that were either *Tsc1* cHets or cKOs were treated for 5 days with rapamycin, as in main Figure 8, and then treatment was ceased for 5 days. Soma size, PV and Kv3.1 expression were assessed in the neocortex and compared to the 5 day vehicle and 5 day rapamycin treated data from main Figure 8. **(a)** Quantification of soma size from each genotype and group: One-way ANOVA ($F_{5, 494} = 74.52$, $P < 0.0001$; cHet veh vs. all cKO groups $p < 0.0001$; cHet 5d rapa vs. all cKO groups $p < 0.0001$; cHet 5d on/off vs. all cKO groups $p < 0.0001$; cKO veh vs cKO 5d rapa $p < 0.0001$). **(b)** Quantification of tdTomato+/Kv3.1+ cell density: One-way ANOVA ($F_{5, 12} = 11.74$, $P < 0.0003$; cKO veh vs. cHet veh $p = 0.0025$, cHet 5d rapa $p = 0.0004$, cHet 5d rapa on/off $p = 0.0003$, cKO 5d rapa $p = 0.0036$ and cKO 5d rapa on/off $p = 0.0433$). **(c)** quantification of tdTomato+/PV+ cell density: One-way ANOVA ($F_{5, 14} = 9.032$, $P = 0.0005$; cKO veh vs. cHet veh $p = 0.0031$, cHet 5d rapa $p = 0.0025$, cHet 5d rapa on/off $p = 0.0017$ and cKO 5d rapa $p = 0.0063$; cKO veh vs. cKO 5d rapa on/off $p = 0.0416$). (AU) arbitrary units. Data are represented as mean ± SEM. n = 3, vehicle and 5d Rapa (75 cells each for soma counts); n = 3, Kv3.1 5d Rapa/5d Off group, n = 4, PV 5d Rapa/5d Off group, (100 cells total/group for soma counts). * $p < 0.05$, ** $p < 0.01$, *** $p < 0.001$, **** $p < 0.0001$. Source data are provided as a Source Data file.

Supplementary Fig. 14

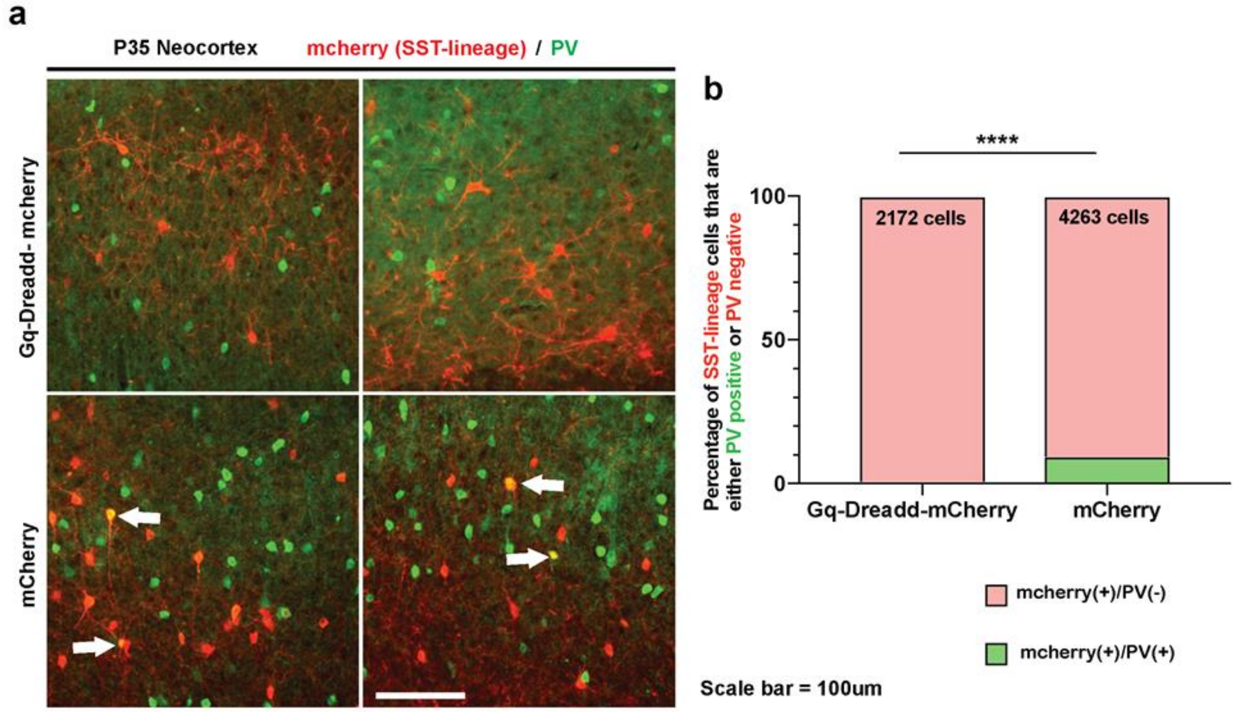

Increasing excitability of wild-type SST CINs decreases PV co-expression

(a) Examples of neocortical images labelled for mCherry (red) and PV expression (green) in experimental group (Gq-Dreadd-mcherry) and control group (mCherry). **(b)** Graph showing the percentage of mCherry(+)/PV(-) and mCherry(+)/PV(+) CINs in the two groups. Chi-squared test, n =3 mice per group. **** p < 0.0001. Source data are provided as a Source Data file.

Supplementary Fig. 15

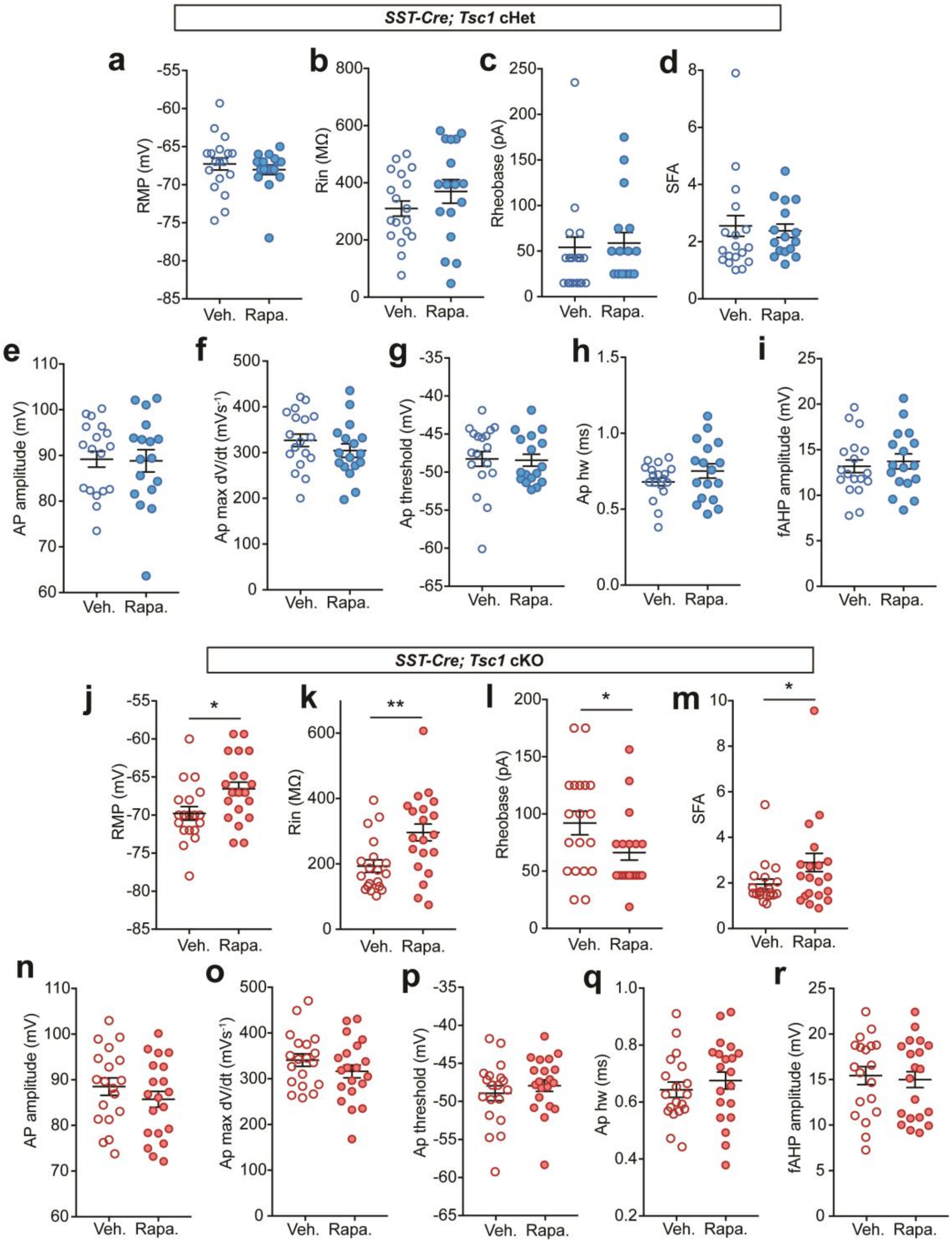

Blocking aberrant mTOR activity with rapamycin affects the subthreshold and firing properties of SST-lineage CINs in *Tsc1* cKO mice

(a, b) Resting membrane potential **(**RMP) and input resistance (R_{in}) of SST-lineage CINs in vehicle (n = 18 from 2 mice) and rapamycin (n = 17 cells from 2 mice) treated cHets were compared: Unpaired two-way t test; RMP:

($t_{33} = 0.73$; R_{in}: $t_{33} = 1.2$). **(c, d)** Rheobase and spike-frequency accommodation (SFA) of SST-lineage CINs in vehicle and rapamycin treated cHets were compared: Unpaired two-way t test; Rheobase: ($t_{33} = 0.28$; SFA: $t_{33} = 0.44$). **(e–i)** Action potential (AP) amplitude, maximum rate of rise (max dV/dt), threshold, half-width and fast afterhyperpolarization (fAHP) amplitude of SST-lineage CINs in vehicle and rapamycin treated cHets were compared: Unpaired two-way t test; AP amplitude: ($t_{33} = 0.11$; max dV/dt: $t_{33} = 0.12$); AP threshold: ($t_{33} = 0.13$); AP half-width: ($t_{33} = 1.3$); fAHP: ($t_{33} = 0.49$). **(j, k)** Resting membrane potential **(**RMP) and input resistance (R_{in}) of SST-lineage CINs in vehicle (n = 19 cells from 2 mice) and rapamycin (n = 20 cells from 2 mice) treated cKOs were compared: Unpaired two-way t test; RMP: ($t_{37} = 2.5$, $P = 0.01$); (R_{in}: $t_{37} = 3.1$, $P = 0.003$). **(l, m)** Rheobase and spike-frequency accommodation (SFA) of SST-lineage CINs in vehicle and rapamycin treated cHets were compared: Unpaired two-way t test; Rheobase: ($t_{37} = 2.1$, $P = 0.039$); SFA: ($t_{37} = 1$, $p = 0.04$). **(n–r)** Action potential (AP) amplitude, maximum rate of rise (max dV/dt), threshold, half-width and fast afterhyperpolarization (fAHP) amplitude of SST-lineage CINs in vehicle and rapamycin treated cHets were compared: Unpaired two-way t test; AP amplitude: ($t_{37} = 1$); max dV/dt: ($t_{37} = 1.2$); AP threshold: ($t_{37} = 0.8$); AP half-width: ($t_{37} = 0.8$); fAHP: ($t_{37} = 0.33$). Data are presented as mean ± S.E.M; * $p < 0.05$, ** $p < 0.01$. Source data are provided as a Source Data file.

Supplementary Fig. 16

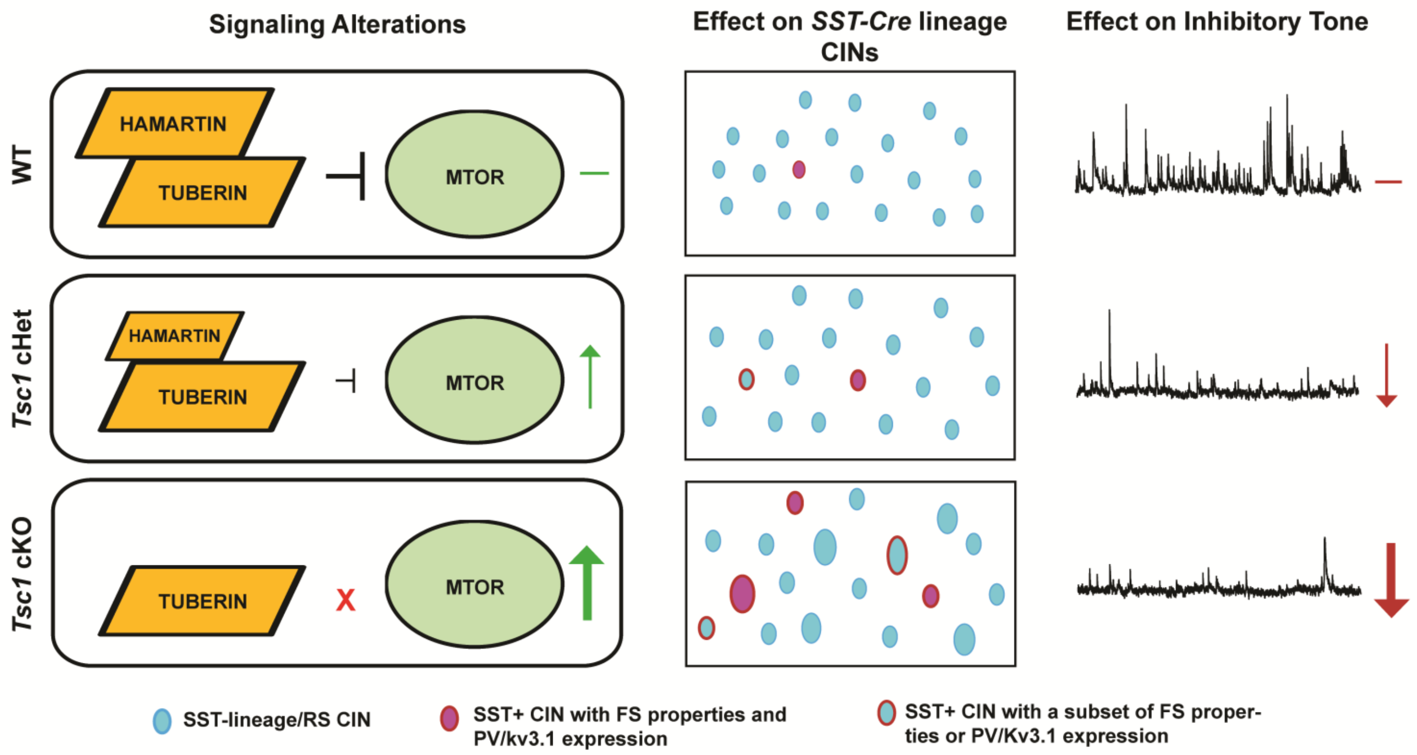

Model describing the effects of *Tsc1* deletion on cellular properties and synaptic output of post-mitotic SST-lineage CINs in *Tsc1* cHets and cKOs

(Left panels) Loss of either one or two *Tsc1* alleles increases MTOR activity with decreased *Tsc1* dosage (green arrows). **(Middle panels)** a subset of SST-lineage CINs increase PV expression and exhibit a continuum of physiological properties associated with FS CINs. While pronounced in the cKO, these changes are subtle in the cHet but observable. Soma size is grossly increased when both copies of *Tsc1* are depleted. **(Right panels)** Loss of *Tsc1* in SST-lineages lowers the amount of inhibition in the neocortex; like other phenotypes, this is pronounced in the cKOs and subtle but observable in the cHets (red arrows).

Supplementary Methods

Animals

Nkx2.1-Cre mice were maintained on a CD-1 background and have been previously described [1]. All animal care and procedures were performed according to the Michigan State University and University of California San Francisco Laboratory Animal Research Center guidelines.

Chemogenetic activation of SST CINs

We injected a Cre-dependent virus expressing the Gq-DREADD (AAV-DJ-Ef1a-DIO-hM3D(Gq)-mCherry, 650 nL) or a control fluorophore (AAV-DJ-Ef1a-DIO-mCherry, 650 nL) into wild-type adult *SST-Cre* mice [2]. We waited 4 weeks for virus expression. To increase the excitability of SST+ CINs, mice were given daily injections of Clozapine-N-oxide (CNO, 3mg/kg i.p.) [3] for 5 days. A day after the last injection, mice were transcardially perfused and brains were processed for PV expression.

Fluorescent *in situ* hybridization

Brain tissues from P30*SST-CRE* WT and *Tsc1* cKOs were perfused and incubated with 4% PFA for 1 hour, followed by incubation in 30% sucrose/PBS for cryoprotection until the day of section. Tissues were embedded in OCT and cryo-sectioned to generate 40um tissue sections.

To generate the *Tsc1* DNA vector and riboprobe, *Tsc1* cDNA was PCR amplified from homemade mouse cDNA library synthesized from P0 CD-1 mouse neocortex using Superscript II.

The following primers were used:

5’ GAGAATCGATAGACAAAGCTGGAGGACTGC

3’ ATATTCTAGATCAAGCCTCTCTTCTGCTGC

ClaI and XbaI restriction enzymes sites were introduced (underlined). The primers target the *Tsc1* floxed regions, which flank exons 16 and 17. Next, the *Tsc1* PCR product and the vector, pSP73 (Promega Cat # P2221), were digested with ClaI and XbaI, and then ligated. The *Tsc1* RNA anti-sense fluorescein-labeled probe was generated by T7 RNA polymerase (Roche) and the Fluorescein labeling kit (Roche) from a NdeI linearized vector, with the size of the probe ~600bp. *Sst* Digoxigenin-labeled riboprobe was kindly provided by Dr. John Rubenstein (original resource T. Lufkin). Fluorescent in situ hybridization was performed as previously described [4]. Imaging was done with a 60x objective (Nikon Apo 1.4 oil) using a Nikon Ti microscope with DS-Ri2 color camera.

Morphological reconstructions

In all our recordings, 0.2–0.3% biocytin was added to the pipette solution. Molecular identity and morphological characteristics of recorded cells were confirmed by tdTomato expression and subsequent staining for PV and biocytin. Slices containing biocytin-filled cells were fixed overnight in a buffered solution containing 4% paraformaldehyde. Slices were rinsed twice in PBS, then blocked and permeabilized for 3hr in PBS with 10% FBS, 0.5% Triton X-100 and 0.05% sodium Azide. Slices were immuno-stained overnight with primary antibody: rabbit anti-PV (Swant, cat. # PV27) diluted 1:1000 in PBS with 0.1% Triton X-100, 10% FBS and 0.025% sodium azide. Slices were washed 2 x 30min in PBS with 0.25% Triton X-100, and 2 x 30min in PBS. Goat anti-rabbit Alexa-488 secondary antibody (1:750, Thermo Fisher, cat. #A-11034) and Streptavidin-647 (1:500, Thermo Fisher, cat. # S-32357) were added with Hoescht 33342 nuclear counterstain (1:2000, Thermo Fisher, cat. # H3570) for 4-6hr at room temperature, then overnight at 4°C. After washing 2 x 30min in PBS with 0.25% Triton X-100, and 2 x 30min in PBS, slices were mounted on Superfrost Plus slides and coverslipped with Dako Fluorescence Mounting Medium (cat. # S3023).

Low-magnification epifluorescent images were taken using a Coolsnap camera (Photometrics) mounted on a Nikon Eclipse 80i microscope using NIS Elements acquisition software (Nikon). Confocal images were taken with 20x air and 40x oil objectives on an Andor Borealis CSU-W1 spinning disk confocal mounted on a Nikon Ti Microscope (UCSF Nikon Imaging Center, NIH S10 Shared Instrumentation grant 1S10OD017993-01A1) and captured with an Andor Zyla sCMOS camera and Micro-Manager software (Open Imaging). Confocal stacks were imported in NeuTube for semi-automated tracing of biocytin-filled neurons [5]. Reconstructions were analyzed using Trees toolbox in MATLAB [6].

Sag and rebound measurements

Sag and rebound were estimated from the voltage responses to hyperpolarizing current injections (-50 to -250 pA, steps of -50 pA). Sag was measured as the percent change between the maximum and steady state voltage change during hyperpolarizing current injections [7]. Rebound slope was measured as the slope of the rebound potential amplitude as a function of the steady-state voltage in response to hyperpolarizing current injections [8].

Supplementary references

1. Xu, Q., Tam, M. & Anderson, S. A. Fate mapping Nkx2.1-lineage cells in the mouse telencephalon. *J. Comp. Neurol.* **506**, 16–29 (2008).

2. Athilingam JC, Ben-Shalom R, Keeshen CM, Sohal VS, Bender KJ. Serotonin enhances excitability and gamma frequency temporal integration in mouse prefrontal fast-spiking interneurons. *eLife* **6**, e31991 (2017).

3. Wang W, Rein B, Zhang F, Tan T, Zhong P, Qin L, et al. Chemogenetic Activation of Prefrontal Cortex Rescues Synaptic and Behavioral Deficits in a Mouse Model of 16p11.2 Deletion Syndrome. *J Neurosci*. **38**, 5939-48 (2018).

4. Duan, X. Krishnaswamy A, Laboulaye MA, Liu J, Peng YR, Yamagata M, Toma K, Sanes JR. Cadherin Combinations Recruit Dendrites of Distinct Retinal Neurons to a Shared Interneuronal Scaffold. *Neuron* **99**, 1145-1154.e6 (2018).

5. Feng, L., Zhao, T. & Kim, J. neuTube 1.0: A New Design for Efficient Neuron Reconstruction Software Based on the SWC Format. *eNeuro* **2**, (2015).

6. Cuntz, H., Forstner, F., Borst, A. & Häusser, M. One rule to grow them all: a general theory of neuronal branching and its practical application. *PLoS Comput. Biol.* **6**, (2010).

7. Brager, D. H., Akhavan, A. R. & Johnston, D. Impaired dendritic expression and plasticity of h-channels in the fmr1(-/y) mouse model of fragile X syndrome. *Cell Rep.* **1**, 225–233 (2012).

8. Malik, R., Dougherty, K. A., Parikh, K., Byrne, C. & Johnston, D. Mapping the electrophysiological and morphological properties of CA1 pyramidal neurons along the longitudinal hippocampal axis. *Hippocampus* **26**, 341–361 (2016).
