# Supplementary material for: Tsc1 represses parvalbumin expression and fast-spiking properties in somatostatin lineage cortical interneurons
Source: Nat Commun. 2019 Nov 1;10:4994. doi: 10.1038/s41467-019-12962-4 (PMC6825152; doi:10.1038/s41467-019-12962-4)
Supplement: Supplementary file 3 — Reporting Summary [file 41467_2019_12962_MOESM3_ESM.pdf]

nature research

Corresponding author(s): Vikaas S. Sohal
Daniel Vogt

Last updated by author(s): Sep 29, 2019

Reporting Summary

Nature Research wishes to improve the reproducibility of the work that we publish. This form provides structure for consistency and transparency in reporting. For further information on Nature Research policies, see Authors & Referees and the Editorial Policy Checklist .

Statistics

For all statistical analyses, confirm that the following items are present in the figure legend, table legend, main text, or Methods section.

n/a	Confirmed	
☐	☒	The exact sample size (n) for each experimental group/condition, given as a discrete number and unit of measurement
☐	☒	A statement on whether measurements were taken from distinct samples or whether the same sample was measured repeatedly
☐	☒	The statistical test(s) used AND whether they are one- or two-sided<br>*Only common tests should be described solely by name; describe more complex techniques in the Methods section.*
☒	☐	A description of all covariates tested
☐	☒	A description of any assumptions or corrections, such as tests of normality and adjustment for multiple comparisons
☐	☒	A full description of the statistical parameters including central tendency (e.g. means) or other basic estimates (e.g. regression coefficient) AND variation (e.g. standard deviation) or associated estimates of uncertainty (e.g. confidence intervals)
☐	☒	For null hypothesis testing, the test statistic (e.g. F, t, r) with confidence intervals, effect sizes, degrees of freedom and P value noted<br>*Give P values as exact values whenever suitable.*
☒	☐	For Bayesian analysis, information on the choice of priors and Markov chain Monte Carlo settings
☒	☐	For hierarchical and complex designs, identification of the appropriate level for tests and full reporting of outcomes
☒	☐	Estimates of effect sizes (e.g. Cohen's d, Pearson's r), indicating how they were calculated

Our web collection on statistics for biologists contains articles on many of the points above.

Software and code

Policy information about availability of computer code

Data collection	Confocal imaging: NIS Elements acquisition software<br>Compound fluorescent microscope imaging: Leica LAS-X software<br>Ephys data: pClamp 10<br>Single neuron model: NEURON + Python<br>Neuronal Morphology: NeuTube
Data analysis	Confocal images: Fiji/ImageJ<br>Compound fluorescent microscope imaging: Fiji/ImageJ<br>Ephys data: pClamp 10 and Igor Pro 7<br>Neuronal Morphology: Trees Toolbox in MATLAB<br>Statistical analysis: Prism 6 andPrism 8

For manuscripts utilizing custom algorithms or software that are central to the research but not yet described in published literature, software must be made available to editors/reviewers. We strongly encourage code deposition in a community repository (e.g. GitHub). See the Nature Research guidelines for submitting code & software for further information.

Data

Policy information about availability of data

All manuscripts must include a data availability statement. This statement should provide the following information, where applicable:

- Accession codes, unique identifiers, or web links for publicly available datasets
- A list of figures that have associated raw data
- A description of any restrictions on data availability

The data and computer scripts included in this study are available from the corresponding authors upon request.

Field-specific reporting

Please select the one below that is the best fit for your research. If you are not sure, read the appropriate sections before making your selection.

- [x] Life sciences
- [ ] Behavioural & social sciences
- [ ] Ecological, evolutionary & environmental sciences

For a reference copy of the document with all sections, see nature.com/documents/nr-reporting-summary-flat.pdf

Life sciences study design

All studies must disclose on these points even when the disclosure is negative.

Sample size	Sample sizes were selected based on data from similar studies in the literature.
Data exclusions	No data were excluded from the analysis
Replication	All the experimental findings were reliably reproduced and the number of replicates are indicated in the corresponding figure legends
Randomization	All animals in a mouse litter were used, there was no selection bias to discard mouse samples
Blinding	Experimenters were blinded to the genotype of the animals during acquisition and analysis

Reporting for specific materials, systems and methods

We require information from authors about some types of materials, experimental systems and methods used in many studies. Here, indicate whether each material, system or method listed is relevant to your study. If you are not sure if a list item applies to your research, read the appropriate section before selecting a response.

Materials & experimental systems

n/a	Involved in the study	
☐	☒	Antibodies
☐	☒	Eukaryotic cell lines
☒	☐	Palaeontology
☐	☒	Animals and other organisms
☒	☐	Human research participants
☒	☐	Clinical data

Methods

n/a	Involved in the study	
☒	☐	ChIP-seq
☒	☐	Flow cytometry
☒	☐	MRI-based neuroimaging

Antibodies

Antibodies used

Primary antibodies:
Mouse anti-Kv3.1b (NeuroMab cat. # 75-041)
Rabbit anti-PV (Swant cat. # PV 27)
Mouse anti-PV (Millipore Sigma, cat. # MAB1572)
Rat anti-SST (Millipore cat. # MAB354)
Rabbit anti-phosphoS6SER240/244, (Cell Signaling Technologies, cat. # 5364)

Secondary antibodies:
Donkey anti-rabbit Alexa 488 (Thermo Fisher, cat. # A-21206)
Donkey anti-mouse Alexa 488 (Thermo Fisher, cat. # A-21202)
Donkey anti-rat Alexa 488 (Thermo Fisher, cat. # A-21208)
Goat anti-rabbit Alexa-488 (Thermo Fisher, cat. #A-11034)
Donkey anti-rabbit Alexa-594 (Thermo Fisher, cat. # A-21207)
Donkey anti-rabbit Alexa-647 (Thermo Fisher, cat. # A-31573)
Donkey anti-mouse Alexa-647 (Thermo Fisher, cat. # A-31571)
Streptavidin-647 (Thermo Fisher, cat. # S-32357)

Validation

All antibodies used were commercially available (see manufacturer's website):
http://neuromab.ucdavis.edu/neuromabs.cfm
https://www.swant.com/?p=products&c=1.3
http://www.emdmillipore.com/US/en/product/Anti-Parvalbumin-Antibody,MM_NF-MAB1572
http://www.emdmillipore.com/US/en/product/Anti-Somatostatin-Antibody-clone-YC7,MM_NF-MAB354

https://www.cellsignal.com/products/primary-antibodies/phospho-s6-ribosomal-protein-ser240-244-d68f8-xp-rabbit-mab/5364

https://www.thermofisher.com/antibody/product/Donkey-anti-Rabbit-IgG-H-L-Highly-Cross-Adsorbed-Secondary-Antibody-Polyclonal/A-21206
https://www.thermofisher.com/antibody/product/Donkey-anti-Mouse-IgG-H-L-Highly-Cross-Adsorbed-Secondary-Antibody-Polyclonal/A-21202
https://www.thermofisher.com/antibody/product/Goat-anti-Rabbit-IgG-H-L-Highly-Cross-Adsorbed-Secondary-Antibody-Polyclonal/A-11034
https://www.thermofisher.com/antibody/product/Donkey-anti-Rat-IgG-H-L-Highly-Cross-Adsorbed-Secondary-Antibody-Polyclonal/A-21208
https://www.thermofisher.com/antibody/product/Donkey-anti-Rabbit-IgG-H-L-Highly-Cross-Adsorbed-Secondary-Antibody-Polyclonal/A-21207
https://www.thermofisher.com/antibody/product/Donkey-anti-Rabbit-IgG-H-L-Highly-Cross-Adsorbed-Secondary-Antibody-Polyclonal/A-31573
https://www.thermofisher.com/antibody/product/Donkey-anti-Mouse-IgG-H-L-Highly-Cross-Adsorbed-Secondary-Antibody-Polyclonal/A-31571
https://www.thermofisher.com/order/catalog/product/S32357

The PV and SST antibodies do not stain any tissue in Lhx6 KO mouse brains, a transcription factor that is necessary for their emergence. Kv3.1b is restricted to PV+ CINs. We provide a supplementary figure showing that the Kv3.1b antibody used detects protein in PV+ CINs. The pS6 antibody used has been highly used and validated and the manufacturer's website details this information.

Eukaryotic cell lines

Policy information about cell lines

Cell line source(s)	HEK293T from ATCC
Authentication	Cells were not authenticated and this is not necessary for the procedures in this manuscript, i.e. lentiviral production
Mycoplasma contamination	Cell lines were not tested for Mycoplasma contamination
Commonly misidentified lines (See ICLAC register)	n.a.

Animals and other organisms

Policy information about studies involving animals; ARRIVE guidelines recommended for reporting animal research

Laboratory animals	mus musculus, mixed background (C57/BL6, CD-1 strain), both male and female mice were used. Age is reported in the results and methods sections.
Wild animals	n/a
Field-collected samples	n/a
Ethics oversight	All animal care and procedures were performed according to the Michigan State University and University of California San Francisco Laboratory Animal Research Center guidelines.

Note that full information on the approval of the study protocol must also be provided in the manuscript.
