# Supplementary material for: Tsc1 represses parvalbumin expression and fast-spiking properties in somatostatin lineage cortical interneurons
Source: Nat Commun. 2019 Nov 1;10:4994. doi: 10.1038/s41467-019-12962-4 (PMC6825152; doi:10.1038/s41467-019-12962-4)
Supplement: Supplementary file 2 — Peer Review [file 41467_2019_12962_MOESM2_ESM.pdf]

Reviewers' comments:

Reviewer #1 (Remarks to the Author):

This paper studies the effect of Tsc1 loss of function on cortical interneuron characteristics. The remarkable finding is presented that Tsc1 LOF in maturing somatostatin-expressing interneurons results in their upregulation of KV3.1, resulting in "fast-spiking" characteristics not generally seen in SST interneurons. This is an important finding, with both clinical and preclinical relevance, and the paper is well presented.

Comments.

First, although PV has been used as a marker of a cortical gabaergic interneuron subgroup, is known to be partially activity dependent and for its levels to be modified during early postnatal development by various factors such as BDNF. While the emphasis on PV is understandable given the literature, the truly remarkable finding in the paper is not upregulation of PV in SST-expressing cells, but the conversion of some SST interneurons to the "fast-spiking" firing characteristic in response to injected current, and the apparently causative upregulation of Kv3.1. For this reason this review suggests clearly explaining the relationship between kv3.1, fast-spiking, and PV in the intro, then referring to the characteristic change as one to FS rather than the emphasis on PV per se (except in the PV data section).

Along the same lines, it is a semantic issue, but the authors refer to their findings as showing that "we propose that the choice between SST+ and PV+ cell fate and function is mediated in part by non-transcriptional processes". If the authors choose to use the word "fate" they should define what they mean by this term in this context. The cells in question maintain SST expression and dendritic targeting--hence one could argue that the loss of Tsc1 results in their acquiring a mixture of characteristics not normally present in neocortical interneurons (but known to occur in a subclass of hippocampal ones). Then is the definition of the word "fate' here the same as the word "characteristic"?

Another problem is with the use of the term "specification" in the discussion. Classically, a cell's fate is "specified" when it maintains some combination of fate-defining characteristics when placed in to a "neutral environment". Under that definition, key neuronal properties, including PV expression itself, are likely never "specified" since they require external developmental influences and, at least for PV, are affected by activity after maturation is complete. Will a PV cell express PV or fast-spike if grown without excitatory inputs or depolarizing conditions that mimic that input?

To this end, this paper is an excellent opportunity, within the confines of space in this format, to indicate that this paper highlights the conceptual challenges to applying concepts of fate specification that evolved in other systems to neurons. Or, this reviewer suggests that the word "fate" should be avoided, in favor of the word "characteristic".

A related question, with their system in place it would not be difficult to use an AAV with a Cre dependent chemogenetic upregulator of activity in SST-interneurons to show whether enhancing SST activity alone without mTOR manipulation can induce PV expression in these cells. Such a finding does not get at the intriguing question on the mechanism by which upregulated mTOR signaling results in Kv3.1 expression, but could at least demonstrate clearly that PV per se is a plastic marker not a stable marker of a defined fate, as implied by many papers showing PV downregulation but not cell loss in response to metabolic challenge or excitatory input loss.

Finally, another relatively simple experiment that could enhance the paper--do neocortical SST interneurons natively express lower levels of pS6 kinase than neocortical PV interneurons?

If so, perhaps we should be thinking not of PV versus SST fate, but mTOR high versus mTOR low fates...

Reviewer #2 (Remarks to the Author):

In this manuscript, the authors generate a conditional loss of Tsc1 (resulting in decreased mTor signaling) in SSTCre mice to specifically knockout this gene in MGE-derived SST+/regular spiking interneurons. Based on neurochemical and detailed e-phys data, the authors conclude that this mutation causes some SST+ RS cells to adopt PV/FS-like properties, with cells falling into a continuum of few/some/most features transitioning to FS cells. The manuscript is well-written, the data on the whole are presented clearly, and the corresponding authors have an extensive history investigating mechanisms regulating interneuron fate and maturation. However, there is some gray area in determining what exactly this genetic perturbation is doing to these cells, and I think a number of experiments could help solidify some of the authors claims and greatly strengthen the manuscript prior to publication.

Major Issues:

1. One issue I'm struggling with is if the effects the authors observe are truly dependent on genetic manipulation of mTor function, or instead if the changes observed in these SST+ cells is primarily a cellular response to the altered increased size of the cell bodies. As the authors note on page 6, increased soma size should result it decreased Rin and corresponding increase in Rheobase, which is what they observe. If one artificially increases cell body size, is that sufficient to induce (some/most?) of the observed changes? One way the authors could investigate this would be to incubate slices from cKO in hypo-osmotic solution for an extended period of time (hours?) and then either 1) record from tomato+ cells to assess ephys properties, or 2) fix slices and characterize changes in cell size with pS6 levels, KV3.1 levels, etc, both within tomato+ cells and in all cells since they should more or less be effected similarly. While I admit this is not the cleanest of experiments, it is a reasonable approach to explore if the observed effects are actually due to genetic manipulation of mTor or a (stress?) response of increasing cell size, in which the mechanical changes will certainly change the leakiness of the cell and likely lead to changes in receptor expression to compensate for this effect.

2. The authors imply that there is a (partial) switch in fate from SST+ RS cells towards PV-like FS cells in the Tsc1 cKO mice. However, their paired recordings (Fig 5) do not indicate a major shift with their outputs onto pyramidal cells and suggest that it 'does not affect their axonal targeting'. The authors don't provide direct data to back up this claim, but it should be available from their recordings. It would be incredibly insightful to include reconstructions of some tomato+ cells to more fully characterize their morphology. Do these cKO cells (and cHets) display axonal morphologies that mimic mature endogenous Martinotti cells? Do they still extend processes up to layer 1? Is there an increase in more typical Basket-like morphologies in the cKO cells? The authors do examine dendritic morphologies in Sup Fig 4, but the morphologies in the biocytin-filled cells are underwhelming. The authors likely have this morphology data from their recordings, and these reconstructions would go a long way towards understanding how the e-phys changes correlate with typical morphological appearances of these cell types and potential fate switches.

3. One defining characteristic of RS cells is sag due to Ih current. I was surprised to not observe sag in the WT traces in Fig 2; did the authors simply not perform enough depolarizing steps to drive/visualize sag? Nor did I see any report of sag throughout the text. As this is a pretty straightforward way to assess a potential shift from sag+ RS cells towards sag-negative FS cells, it would be insightful to include this analysis in the manuscript. If the authors are correct and there is a partial fate-switch occurring, then you would predict a loss of sag in the most FS-like cKO cells.

4. Throughout the text, the authors compare cHet and cKO to WT SST+ cells. It would be useful to also have examples of WT PV/FS cell properties in the text. I'm not recommending that the authors' repeat all ephys experiments on PV+ cells, but in some instances it would be helpful. For example, in figure 2g-h, it would be helpful to compare APs in current injections in standard FS cells vs the WT, cHet and cKO cells. Do the strongest transformed cKO cells truly fire at FS frequencies? This type of integration of FS cell data would aid our understanding in how fully some cKO cells adopt FS properties. The authors present some endogenous PV/FS cell date in Fig 5, it would be nice to sprinkle some more throughout other images to enhance interpretation of these findings.

5. There are some interesting comparisons/contrasts between this manuscript and the authors previous manuscript investigating mTor signaling (Vogt…Rubenstein Cell Reports 2015). The authors briefly note that a similar upregulation of PV occurs in SST+ cells in Nkx2.1Cre mice in Sup Fig 2. In the previous manuscript, they noted that there was a significant loss (~50%) in SST+ cells when mTor was disrupted in Nkx2.1Cre;Pten cKO mice. Do the authors see a similar loss of SST+ cells in the Nkx2.1Cre;Tsc1 cKO mice in this study? It would be interesting to note if a similar cell loss is observed using 2 different cKO models to perturb mTor function. Notably, PV upregulation was not observed in SSTcre;Pten cKO mice in the previous study (Fig 2 in that paper), in stark contrast to this report (it's unclear to me if cell swelling was observed in these SSTcre;Pten cKO mice). Do the authors have insight into this contrast based on differential function of Tsc1 and Pten in regulating mTor function? The authors have a nice opportunity to compare and contrast these similar studies, but they do not take advantage of this in the discussion.

6. The rapamycin results are very intriguing, that the soma size and %PV expression in the cKO can be partially rescued. It would be helpful to know if KV3.1 expression reverts back to minimal levels in SSTCre cells after this 5-day treatment, which would be predicted (in part) based on fewer cKO cells displaying FS-like properties. It would also be of interest to know if this rescue is reversible. If the investigators examined brains ~5 days after cessation of rapamycin treatment, would the cKO cells return to the larger size and increased %PV expression. I'd also be curious to investigate if the observed defects arise during development or if similar effects could be obtained by Tsc1 cKO in juvenile/adult mice. Maybe using AAVs to KO Tsc1 in juvenile mice could be utilized, but this is a different type of developmental question that may be beyond the scope of this study.

Minor Issues:

7. At times, I feel that some of the authors' claims are stronger than the data supports. For example, on p. 5, the authors claim that '…these data suggest that Tsc1 represses PV expression in SST-lineages…'. Since only ~13% of SSTCre cells upregulate PV protein and the authors don't directly assess repression per se, I feel this strong a claim is tenuous. The title of the first section, 'Loss of Tsc1 causes ectopic expression of PV…' is a more accurate assessment of what their data demonstrates. I feel that stressing Tsc1 repressing features at various points (including the manuscript title) could be altered to better represent the authors findings.

8. In Figure 1, the X-axis labels should be moved to the Y-axis to more accurately reflect what the graphs are displaying, with WT, cHet and cKO on the X-axis as with other graphs throughout the manuscript.

9. In Sup Fig 1, the authors depict images showing upregulation of pS6 in tdTomato cells, but there is no quantification with this data. Do more Tom+ cells express pS6, or do cells simply express higher levels of pS6? The proper quantification is shown in Sup Fig 7, it would be nice to see that quantification here as well.

10. The authors note that there is no change in tomato+ cell numbers in the cKO mice, but what about cell numbers in specific layers (or at least superficial vs. deep)? It would be helpful to know if any changes in layering occur, as this could provide insight into functional changes if there are

alterations in SST+ cell migration/layering.

11. In Sup Fig 7 images, it appears that in the Tsc1 cKO mice, Tom+ cells express lower levels of pS6 rather than a loss of pS6 expression. Most (all?) red cells in the lower right panel still appear yellow indicating they are in fact pS6+. ¬¬This image does not seem to support the authors' claim in the adjacent bar graph of a strong reduction of pS6 signal in rapamycin condition. Is this just due to image processing, or non-optimal representative image? This point should be clarified.

12. In the model in Sup fig 9, the authors should distinguish between PV+ cells and FS-like properties, as these do not cleanly overlap. Only ~13% of SSTcre cKO cells express PV (Fig 1J), but this model makes it seem that it's closer to 50%. Maybe make a different color for PV+ and FS properties?

13. The implication sprinkled throughout the text is that the Tsc1 cKO cells are on a continuum that adapt few/some/many properties of normal PV cells. The authors do attempt to link some of these features together (Fig 2, Sup fig 4). In addition to these insights, I was curious if the cKO cells that upregulate PV tend to have larger cell bodies than non-PV cells? Is there a correlation between soma size and PV expression?

14. It's surprising that there is a small but significant increase in SST+/PV+ cells in the SSTCre cHet but not in the Nkx2.1Cre cHet in Sup Fig 2. Do the authors have an idea as to why this is the case?

Reviewer #3 (Remarks to the Author):

In this manuscript, Malik et al. describe the results of Tsc1 deletion in somatostatin positive interneurons in a genetic mouse model utilizing the Cre/lox system. Both heterozygous and homozygous mutation causes a subset of the somatostatin positive cells (regular spiking) to take on a parvalbumin positive interneuron like phenotype (fast spiking). The fast spiking nature of the mutant neurons is linked to overexpression of Kv3.1. However, despite the fast spiking qualities, the mutant neurons have decreased inhibitory synaptic output, potentially contributing to decreased inhibitory tone in this disease model. Finally, a short chronic treatment with rapamycin is able to decrease the expression of parvalbumin-like phenotypes in the mutant somatostatin lineage neurons.

Overall, the study addresses an important question (role of Tsc1 in inhibitory neurons); data are well-presented; statistical analyses appear appropriate; and the result that there is decreased inhibitory tone in cKO mice is potentially translationally significant.

There are a few moderate issues that need to be addressed to increase the impact of the manuscript.

Specific comments:
It is clear that TSC disease is due to a combination of haploinsufficiency and loss of heterozygosity (LOH). One question often discussed in the field is whether a certain manifestation is due to heterozygous state or due to LOH. I am not convinced that there is much change in the Tsc1 heterozygous state in inhibitory neurons. Cell size in Fig 1h is not changed in het cells. Rheobase in Fig 2f is not changed in het cells, etc. In Fig 4, reduced inhibitory synaptic output of the SST+ neurons is almost all specific to biallelic loss. More importantly, rapamycin does not make any significant effect on the phenotypes in Tsc1 het neurons (Fig 7), so the comments on the effect of Tsc1 heterozygosity should be moderated.

Abstract says "These changes also occur when only one allele of Tsc1 is deleted, making these findings relevant to individuals with TS."
Very few of the changes actually occur in the heterozygous state. This statement should be removed or revised.

Page 4: "SST-Cre-lineage cHet and cKO cells in the neocortex had elevated levels of ribosomal subunit S6 phosphorylated at Serines 240 and 244 (Supplementary Figs. 1e-g), indicating increased MTOR activity."
There is no quantification in Supplementary Figs. 1e-g to justify this statement.

Page 5: "A similar phenotype of ectopic PV expression in SST+ CINs after Tsc1 deletion was observed in Nkx2.1-Cre; Tsc1 conditional mutants (Fig. S2)."
According to the figure, Tsc1 hets do not differ from WT. So, this appears different than the findings in SST-Cre mice.

Page 6: "loss of Tsc1 decreased the input resistance (Rin) and produced a corresponding increase in the rheobase (current threshold) of CINs in cHets and cKOs (Figs. 2c, f)."
The only significant differences according to the figures (Fig 1c and 1f) are between WT and cKO, not hets.

Fig 1q: the authors used 3 mice in each group and report a significant difference between 0% and 2% with this cohort size. The SEM shown on the graph in this panel seems surprisingly small for n of 3. Could you please double-check that n of animals (not n of cells) was used for this graph?

Were the expression of any other ion channels besides Kv3.1 investigated? Please explain the rationale for this specific choice better.

In Figure 3b, were the Kv3.1 + tdTomato expressing cells co-localized with increased PV expression?

Rapamycin dose used in this study (10mg/kg IP every day) is a very high dose based on the PK of rapamycin in the brain. It would be helpful to include a reason why this high dose was chosen.

Please include a discussion the recent paper by Zhao and Yoshii (PMID: 30683131), which does not find a phenotype in the selective deletion of Tsc1 from either PV or SST cells.

Minor comments:
Please use the conventional abbreviation for Tuberous Sclerosis Complex (TSC) for this disorder. TS is not commonly used.

The role of the mTOR complex in transcriptional regulation as it relates to what is known about patterning of the MGE should be briefly discussed. For example, does mTOR dysregulation affect Lhx6 expression (mentioned in the introduction)?

Line 3 Page 4- missing "we" after "To test this,"

Response to reviewers' comments

We thank the reviewers for their positive response, and their critical and constructive comments on our manuscript. In response to the reviewers' comments, we have made significant changes to the manuscript specifically addressing the reviewers' concerns, also performing additional experiments and analyses as necessary. We believe that these changes overall have significantly improved and strengthened the manuscript.

Please find below our point-by-point responses (*italicized*) to the reviewers' comments.

Reviewers' comments:

Reviewer #1 (Remarks to the Author):

This paper studies the effect of Tsc1 loss of function on cortical interneuron characteristics. The remarkable finding is presented that Tsc1 LOF in maturing somatostatin-expressing interneurons results in their upregulation of KV3.1, resulting in "fast-spiking" characteristics not generally seen in SST interneurons. This is an important finding, with both clinical and preclinical relevance, and the paper is well presented.

Comments.

First, although PV has been used as a marker of a cortical gabaergic interneuron subgroup, is known to be partially activity dependent and for its levels to be modified during early postnatal development by various factors such as BDNF. While the emphasis on PV is understandable given the literature, the truly remarkable finding in the paper is not upregulation of PV in SST-expressing cells, but the conversion of some SST interneurons to the "fast-spiking" firing characteristic in response to injected current, and the apparently causative upregulation of Kv3.1. For this reason this review suggests clearly explaining the relationship between kv3.1, fast-spiking, and PV in the intro, then referring to the characteristic change as one to FS rather than the emphasis on PV per se (except in the PV data section).

We have revised the introduction section to describe the relationship between Kv3.1 and fast-spiking physiology in PV+ CINs.

Along the same lines, it is a semantic issue, but the authors refer to their findings as showing that "we propose that the choice between SST+ and PV+ cell fate and function is mediated in part by non-transcriptional processes". If the authors choose to use the word "fate" they should define what they mean by this term in this context. The cells in question maintain SST expression and dendritic targeting--hence one could argue that the loss of Tsc1 results in their acquiring a mixture of characteristics not normally present in neocortical interneurons (but known to occur in a subclass of hippocampal ones). Then is the definition of the word "fate' here the same as the word "characteristic"?

Another problem is with the use of the term "specification" in the discussion. Classically, a cell's fate is "specified" when it maintains some combination of fate-defining characteristics when placed in to a "neutral environment". Under that definition, key neuronal properties, including PV expression itself, are likely never "specified" since they require external developmental influences and, at least for PV, are affected by activity after maturation is complete. Will a PV cell express PV or fast-spike if grown without excitatory inputs or depolarizing conditions that mimic that input?

To this end, this paper is an excellent opportunity, within the confines of space in this format, to indicate that this paper highlights the conceptual challenges to applying concepts of fate specification that evolved in other systems to neurons. Or, this reviewer suggests that the word "fate" should be avoided, in favor of the word "characteristic".

These are important points. Based on the reviewer’s suggestion, we have replaced the terms “fate” and “specification” with “programming”. In the revised manuscript, the term “programming” is defined as the combined molecular, cellular and physiological characteristics of CINs. This definition is mentioned in the introduction section.

A related question, with their system in place it would not be difficult to use an AAV with a Cre dependent chemogenetic upregulator of activity in SST-interneurons to show whether enhancing SST activity alone without mTOR manipulation can induce PV expression in these cells. Such a finding does not get at the intriguing question on the mechanism by which upregulated mTOR signaling results in Kv3.1 expression, but could at least demonstrate clearly that PV per se is a plastic marker not a stable marker of a defined fate, as implied by many papers showing PV down-regulation but not cell loss in response to metabolic challenge or excitatory input loss.

Whether SST+ CIN cell fate/programming is affected by changes in activity of these neurons is not known. While circuit activity was not a primary tenet of our study, this is a very important to understand the dynamic regulation of CIN cell programming in adult brains. We conducted the experiment suggested by the reviewer and chemogenetically increased SST CIN activity for 5 days. Wild-type SST-Cre mice expressing either AAV-

DIO-Gq-DREADD-mcherry (treatment group, 3 mice) or AAV-DIO-mcherry (control group, 3 mice) were given daily i.p injections of clozapine-N-oxide (CNO, agonist for DREADDs, 3 mg/Kg dose) for 5 days. A day after the last injection, mice were transcardially perfused and the expression of PV in AAV transduced cells was quantified. As previously noted, a small percentage of SST-Cre lineage (mCherry+) CINs in the control mice co-expressed PV. Interestingly, Gq-DREADD expressing SST+ CINs had significantly lower expression of PV. This suggests that increasing the activity of SST+ CINs decreases the expression of PV in these CINs. This is an important and novel finding and we thank the reviewer for suggesting this experiment. Data from this experiment is shown in Supplementary Fig. 14.

Finally, another relatively simple experiment that could enhance the paper--do neocortical SST interneurons natively express lower levels of pS6 kinase than neocortical PV interneurons? If so, perhaps we should be thinking not of PV versus SST fate, but mTOR high versus mTOR low fates...

This is a very important question and we thank the reviewer for suggesting this analysis. To address this, we compared the pS6 levels in PV+ and SST+ CINs in young adult, P35, wild-type mice. Consistent with the idea the MTOR activity may be involved in some PV/FS programming, ~70% of PV+ CINs have detectable pS6 labeling, compared to just ~20% of SST+ CINs. We believe this a novel observation that strengthens the findings of our manuscript, and demonstrates that in the normal brain, MTOR activity is already elevated preferentially in PV CINs. We thank the reviewer for this insight. Data from this analysis now comprise Fig. 2.

Reviewer #2 (Remarks to the Author):

In this manuscript, the authors generate a conditional loss of Tsc1 (resulting in decreased mTor signaling) in SSTCre mice to specifically knockout this gene in MGE-derived SST+/regular spiking interneurons. Based on neurochemical and detailed e-phys data, the authors conclude that this mutation causes some SST+ RS cells to adopt PV/FS-like properties, with cells falling into a continuum of few/some/most features transitioning to FS cells. The manuscript is well-written, the data on the whole are presented clearly, and the corresponding authors have an extensive history investigating mechanisms regulating interneuron fate and maturation. However, there is some gray area in determining what exactly this genetic perturbation is doing to these cells, and I think a number of experiments could help solidify some of the authors claims and greatly strengthen the manuscript prior to publication.

Major Issues:

1. One issue I'm struggling with is if the effects the authors observe are truly dependent on genetic manipulation of mTor function, or instead if the changes observed in these SST+ cells is primarily a cellular response to the altered increased size of the cell bodies. As the authors note on page 6, increased soma size should result it decreased Rin and corresponding increase in Rheobase, which is what they observe. If one artificially increases cell body size, is that sufficient to induce (some/most?) of the observed changes? One way the authors could investigate this would be to incubate slices from cKO in hypo-osmotic solution for an extended period of time (hours?) and then either 1) record from tomato+ cells to assess ephys properties, or 2) fix slices and characterize changes in cell size with pS6 levels, KV3.1 levels, etc, both within tomato+ cells and in all cells since they should more or less be effected similarly. While I admit this is not the cleanest of experiments, it is a reasonable approach to explore if the observed effects are actually due to genetic manipulation of mTor or a (stress?) response of increasing cell size, in which the mechanical changes will certainly change the leakiness of the cell and likely lead to changes in receptor expression to compensate for this effect.

We thank the reviewer for raising this important question of whether PV expression and FS physiology in SST+ CINs could be caused due to an increase in cell size (and increased mechanical stress on the cell membrane). We took two different approaches to test this possibility–

A) We took advantage of our cKO cells, in which we have a mixed population of both normal and larger soma sizes. We co-labeled SST-Cre; cKO tissue sections (tdTomato+ cells) with either pS6, Kv3.1 or PV and then assessed whether a specific marker correlated with the soma size of tdTomato+ cells. This allowed us to determine if cell size is strongly associated with the expression of any marker. Interestingly, expression of pS6 was strongly correlated with a larger soma size. Similar correlations were, however, not observed with the expression of PV and Kv3.1 (Supplementary Fig. 11 in the revised manuscript). This analysis suggests that an increase in soma size alone does not correlate with expression of PV and Kv3.1. We also compared the input resistances of FS and RS SST+ CINs in cKOs with native PV+ CINs. If larger soma sizes underlie PV expression (and FS physiology) in SST+ CINs, most FS SST+ CINs would have lower input resistances. However, we did not observe this in our analysis (Supplementary Fig. 7b in the revised manuscript). Specifically, many FS SST+ CINs had higher input resistances (in comparison to native PV+ CINs) and not all SST+ CINs with lower input resistance had FS properties.

B) We conducted the experiment suggested by the reviewer (data shown below). We incubated acute slices from wild-type SST-IRES-Cre; Ai14 mice in a hypo-osmotic ACSF solution (270 mOsm) for 3–4 hrs (to allow enough time for cellular signaling events and protein expression to occur). Patch clamp recordings were

obtained from tdTomato expressing SST+ CINs in slices incubated in 270 mOsm ACSF. The physiological properties of these CINs were compared to properties of SST+ CINs in slices kept in standard ACSF (300 mOsm). The input resistance of SST+ CINs in 270 mOsm ACSF was slightly smaller (not significant) in comparison to CINs in 300 mOsm ACSF suggesting that the incubation in low osmolarity ACSF increased cell size. Interestingly, incubation in 270 mOSm ACSF did not change the firing properties of SST+ CINs. It is possible that increasing the incubation period and lowering the osmolarity further might affect the firing properties of SST+ CINs. However, drastic changes in osmolarity and longer incubation times are not possible in acute slice preparations. Overall, the findings from this experiment together with the cell-size correlation analysis (mentioned above) suggest that mechanical stress caused due to increased cell size is an unlikely contributor to changes in physiological properties of SST+ CINs.

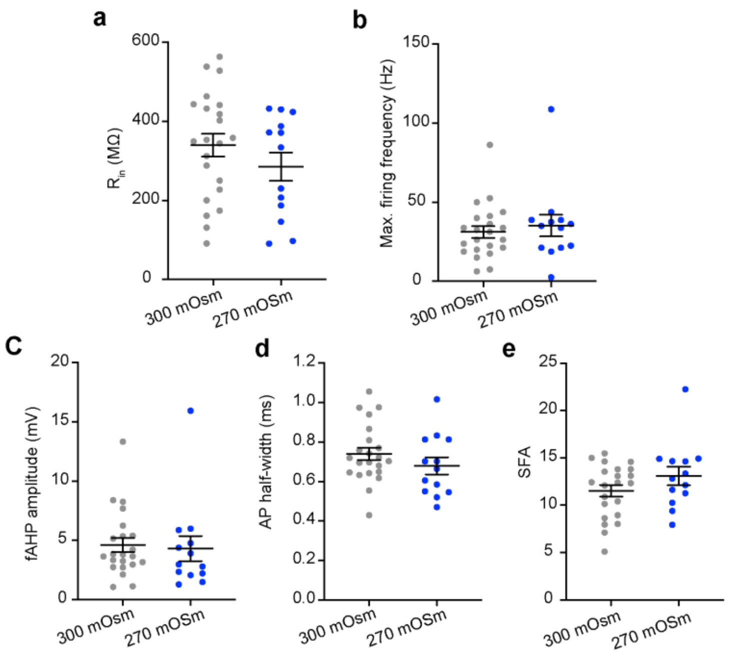

Incubation in low osmolarity ACSF does not change the physiological properties of SST+ CINs

(a) Comparison of input resistance (R_{in}) of SST+ CINs recorded from acute cortical slices incubated in low osmolarity ACSF (270 mOsm, blue circles) and from cortical slices incubated in standard ACSF (300 mOsm, grey circles) (t_{33} = 1.15 p = 0.25. **(b–e)** Same as **a** for maximum firing frequency (t_{33} = 0.57, p = 0.37) **(b)**, fAHP amplitude (t_{33} = 1.43, p = 0.16) **(c)**, AP half-width (t_{33} = 1.6, p = 0.25) **(d)** and SFA index (t_{33} = 0.27, p = 0.78) **(e)**. Two-way unpaired t-test; data are presented as mean ± S.E.M.

2. The authors imply that there is a (partial) switch in fate from SST+ RS cells towards PV-like FS cells in the Tsc1 cKO mice. However, their paired recordings (Fig 5) do not indicate a major shift with their outputs onto pyramidal cells and suggest that it 'does not affect their axonal targeting'. The authors don't provide direct data to back up this claim, but it should be available from their recordings. It would be incredibly insightful to include reconstructions of some tomato+ cells to more fully characterize their morphology. Do these cKO cells (and cHets) display axonal morphologies that mimic mature endogenous Martinotti cells? Do they still extend processes up to layer 1? Is there an increase in more typical Basket-like morphologies in the cKO cells? The authors do examine dendritic morphologies in Sup Fig 4, but the morphologies in the biocytin-filled cells are underwhelming. The authors likely have this morphology data from their recordings, and these reconstructions

would go a long way towards understanding how the e-phys changes correlate with typical morphological appearances of these cell types and potential fate switches.

We agree that comparisons of axonal reconstructions of the recorded neurons would allow us to directly test whether loss of Tsc1 in SST+ CINs and a switch in the physiology from RS to FS affects their axonal targeting. Our previous dendritic morphology analysis was obtained from images limited to layer 5. We reimaged a subset of SST+ CINs in WT and cKOs to quantify the morphological properties spanning all layers of the cortex. In our new analysis, we have divided the cKO CINs into RS and FS CINs (Supplementary Fig. 4 in the revised manuscript). Similar to our previous results, cKO SST+ CINs had more elaborate dendritic branching (observed for both FS and RS SST+ CINs). However, the axonal branching and axonal area in layer 1 were not different between the WT and cKO CINs. Specifically, axon morphology of FS and RS SST+ CINs in cKOs was not different. Together, these results corroborate our findings from the dual patch clamp analysis and show that loss of Tsc1 and shift to FS physiology does not affect the axonal targeting of SST+ CINs.

3. One defining characteristic of RS cells is sag due to Ih current. I was surprised to not observe sag in the WT traces in Fig 2; did the authors simply not perform enough depolarizing steps to drive/visualize sag? Nor did I see any report of sag throughout the text. As this is a pretty straightforward way to assess a potential shift from sag+ RS cells towards sag-negative FS cells, it would be insightful to include this analysis in the manuscript. If the authors are correct and there is a partial fate-switch occurring, then you would predict a loss of sag in the most FS-like cKO cells.

We analyzed the effects of Tsc1 deletion on sag and rebound of SST+ CINs and also compared these Ih sensitive measurements in SST+ CINs to WT FS/PV+ CINs in the revised manuscript (Supplementary Fig. 7d–f). Our new analysis shows that WT SST+ CINs have significantly larger sag and rebound in comparison to WT PV+ CINs. However, Tsc1 deletion in cHets and cKOs does not affect sag and rebound of SST+ CINs. Further, SST+ CINs with FS firing properties did not have PV-like lower sag and rebound values. This suggests that while Tsc1 deletion causes a subset of SST+ CINs to acquire FS firing properties (likely due to increased expression of Kv3.1 channels), this deletion does not affect Ih current and Ih sensitive voltage sag and rebound.

4. Throughout the text, the authors compare cHet and cKO to WT SST+ cells. It would be useful to also have examples of WT PV/FS cell properties in the text. I'm not recommending that the authors' repeat all ephys experiments on PV+ cells, but in some instances it would be helpful. For example, in figure 2g-h, it would be helpful to compare APs in current injections in standard FS cells vs the WT, cHet and cKO cells. Do the

strongest transformed cKO cells truly fire at FS frequencies? This type of integration of FS cell data would aid our understanding in how fully some cKO cells adopt FS properties. The authors present some endogenous PV/FS cell date in Fig 5, it would be nice to sprinkle some more throughout other images to enhance interpretation of these findings.

We thank the reviewer for pointing this out. We have made new figures (Supplementary Figs. 6 and 7) showing the comparison of subthreshold, firing properties and single AP properties of WT FS cells with both RS and FS SST+ CINs. We have added firing frequency data from WT FS (recorded from PV-Cre mice) in Fig. 3h. Within these figures, recorded SST+ cells with FS properties are denoted as black filled circles. Together, these plots show that many FS SST+ CINs have maximal firing frequencies, AP half-widths and fAHP amplitudes similar to WT FS (PV+) CINs.

5. There are some interesting comparisons/contrasts between this manuscript and the authors previous manuscript investigating mTor signaling (Vogt…Rubenstein Cell Reports 2015). The authors briefly note that a similar upregulation of PV occurs in SST+ cells in Nkx2.1Cre mice in Sup Fig 2. In the previous manuscript, they noted that there was a significant loss (~50%) in SST+ cells when mTor was disrupted in Nkx2.1Cre; Pten cKO mice. Do the authors see a similar loss of SST+ cells in the Nkx2.1Cre; Tsc1 cKO mice in this study? It would be interesting to note if a similar cell loss is observed using 2 different cKO models to perturb mTor function. Notably, PV upregulation was not observed in SSTcre;Pten cKO mice in the previous study (Fig 2 in that paper), in stark contrast to this report (it's unclear to me if cell swelling was observed in these SSTcre;Pten cKO mice). Do the authors have insight into this contrast based on differential function of Tsc1 and Pten in regulating mTor function? The authors have a nice opportunity to compare and contrast these similar studies, but they do not take advantage of this in the discussion.

We have added text in the discussion section to compare our results with the Pten paper. We agree this was a missed opportunity and have added key points relevant to this manuscript. In addition, we have an ongoing project examining the Nkx2.1-Cre; Tsc1 mutant mice and do not want to include too much data in the discussion on this project. This is mostly due to a multitude of phenotypes in these mice, including many manifesting outside of the brain. While we do not yet understand the mechanisms, we feel it could be premature to assume the unique Nkx2.1-Cre brain phenotypes are due solely to deletion of Tsc1 in this lineage and need time to figure this out.

6. The rapamycin results are very intriguing, that the soma size and %PV expression in the cKO can be partially rescued. It would be helpful to know if KV3.1 expression reverts back to minimal levels in SST Cre

cells after this 5-day treatment, which would be predicted (in part) based on fewer cKO cells displaying FS-like properties. It would also be of interest to know if this rescue is reversible. If the investigators examined brains ~5 days after cessation of rapamycin treatment, would the cKO cells return to the larger size and increased %PV expression.

We have now added Kv3.1 data to these experiments. Notably, Kv3.1 levels parallel the changes in PV expression. We also looked at these markers at 5 days after cessation of rapamycin treatment. Interestingly, soma sizes were decreased even 5 days after stopping rapamycin treatment. However, the PV and Kv3.1 levels were increased suggesting that expression of these proteins dynamically changes with MTOR activity. This also suggests that the rapamycin mediated rescue of PV expression is reversible. These data now comprise new Sup. Fig. 11.

7. I'd also be curious to investigate if the observed defects arise during development or if similar effects could be obtained by Tsc1 cKO in juvenile/adult mice. Maybe using AAVs to KO Tsc1 in juvenile mice could be utilized, but this is a different type of developmental question that may be beyond the scope of this study.

We agree that it is important to understand whether the effects in Tsc1 cKOs observed in our study might be linked to developmental changes. Also, it would be interesting to compare the effects of conditional Tsc1 deletion in juvenile/adult mice with the observations in our study. These are important questions for future work and are beyond the scope of the present study.

Minor Issues:

8. At times, I feel that some of the authors' claims are stronger than the data supports. For example, on p. 5, the authors claim that '…these data suggest that Tsc1 represses PV expression in SST-lineages…'. Since only ~13% of SSTCre cells upregulate PV protein and the authors don't directly assess repression per se, I feel this strong a claim is tenuous. The title of the first section, 'Loss of Tsc1 causes ectopic expression of PV…' is a more accurate assessment of what their data demonstrates. I feel that stressing Tsc1 repressing features at various points (including the manuscript title) could be altered to better represent the authors findings.

We thank the reviewer for pointing this out. We have changed the text on p. 5 from '…these data suggest that Tsc1 represses PV expression in SST-lineages…' to '…these data suggest that Tsc1 deletion causes PV expression in a subset of SST-lineage CINs,…'.

9. In Figure 1, the X-axis labels should be moved to the Y-axis to more accurately reflect what the graphs are displaying, with WT, cHet and cKO on the X-axis as with other graphs throughout the manuscript.

This has been changed in Fig. 1.

10. In Sup Fig 1, the authors depict images showing upregulation of pS6 in tdTomato cells, but there is no quantification with this data. Do more Tom+ cells express pS6, or do cells simply express higher levels of pS6? The proper quantification is shown in Sup Fig 7, it would be nice to see that quantification here as well.

We have added this quantification to Sup Fig. 1

11. The authors note that there is no change in tomato+ cell numbers in the cKO mice, but what about cell numbers in specific layers (or at least superficial vs. deep)? It would be helpful to know if any changes in layering occur, as this could provide insight into functional changes if there are alterations in SST+ cell migration/layering.

We quantified the laminar distribution of each genotype but did not find any significant differences. These data are new Sup. Fig. 2

12. In Sup Fig 7 images, it appears that in the Tsc1 cKO mice, Tom+ cells express lower levels of pS6 rather than a loss of pS6 expression. Most (all?) red cells in the lower right panel still appear yellow indicating they are in fact pS6+. ¬¬This image does not seem to support the authors' claim in the adjacent bar graph of a strong reduction of pS6 signal in rapamycin condition. Is this just due to image processing, or non-optimal representative image? This point should be clarified.

We thank the reviewer for noticing this. We have gone through all the immunofluorescent images and do see several cells in this group with lower expression that were not counted before. To err on the side of caution, we have recounted these cells and included any with low pS6 staining. There is still a significant decrease in pS6 levels and we revised the graph to encompass these new counts.

13. In the model in Sup fig 9, the authors should distinguish between PV+ cells and FS-like properties, as these do not cleanly overlap. Only ~13% of SSTcre cKO cells express PV (Fig 1J), but this model makes it seem that it's closer to 50%. Maybe make a different color for PV+ and FS properties?

We thank the reviewer for suggesting changes to the model to better depict the findings in our study. Based on the suggestions, we have revised this Figure; it is now Sup. Fig. 16.

14. The implication sprinkled throughout the text is that the Tsc1 cKO cells are on a continuum that adapt few/some/many properties of normal PV cells. The authors do attempt to link some of these features together (Fig 2, Sup fig 4). In addition to these insights, I was curious if the cKO cells that upregulate PV tend to have larger cell bodies than non-PV cells? Is there a correlation between soma size and PV expression?

Please see our response to major point # 1; we did not observe a correlation between cell-size and PV or Kv3.1 expression in cKO SST+ CINs. We have also added new Sup. Fig. 10 that investigates the correlation between cell size and marker expression.

15. It's surprising that there is a small but significant increase in SST+/PV+ cells in the SSTCre cHet but not in the Nkx2.1Cre cHet in Sup Fig 2. Do the authors have an idea as to why this is the case?

While we are not entirely sure, the Nkx2.1-Cre mouse fails to recombine in the dorsal MGE, leading to ~30% of MGE derived CINs that will not delete Tsc1. In contrast, the SST-Cre line recombines in nearly all the SST-lineage CINs. One possibility is that the dorsal MGE population that Nkx2.1-Cre can't target are more susceptible to Tsc1 loss and we were not able to measure these cells in the cHet. Alternatively, the numbers of co-labeled cells are already small in the Nkx2.1-Cre and SST-Cre cHets. This could lead to more variability and more difficulty in determining significance. One issue we found is that the Nkx2.1-Cre graph was not scaled the same way as the SST-Cre graph; this has been corrected. We also checked over the numbers and found the following: In the SST-Cre experiments, there are 10x as many dual labelled cells in the cHet and ~57x more dual labeled cells in the cKO, compared to WT. Nkx2.1 dual labeled cHet cells are 3x more and cKO cells are 224x more than WT. Overall, there is an increase in the cHets using both Cre lines, although we are cognizant that they are not proportionally equal.

Reviewer #3 (Remarks to the Author):

In this manuscript, Malik et al. describe the results of Tsc1 deletion in somatostatin positive interneurons in a genetic mouse model utilizing the Cre/lox system. Both heterozygous and homozygous mutation causes a subset of the somatostatin positive cells (regular spiking) to take on a parvalbumin positive interneuron like phenotype (fast spiking). The fast spiking nature of the mutant neurons is linked to overexpression of Kv3.1. However, despite the fast spiking qualities, the mutant neurons have decreased inhibitory synaptic output, potentially contributing to decreased inhibitory tone in this disease model. Finally, a short chronic treatment with rapamycin is able to decrease the expression of parvalbumin-like phenotypes in the mutant somatostatin lineage neurons.

Overall, the study addresses an important question (role of Tsc1 in inhibitory neurons); data are well-presented; statistical analyses appear appropriate; and the result that there is decreased inhibitory tone in cKO mice is potentially translationally significant.

There are a few moderate issues that need to be addressed to increase the impact of the manuscript.

Specific comments:
1. It is clear that TSC disease is due to a combination of haploinsufficiency and loss of heterozygosity (LOH). One question often discussed in the field is whether a certain manifestation is due to heterozygous state or due to LOH. I am not convinced that there is much change in the Tsc1 heterozygous state in inhibitory neurons. Cell size in Fig 1h is not changed in het cells. Rheobase in Fig 2f is not changed in het cells, etc. In Fig 4, reduced inhibitory synaptic output of the SST+ neurons is almost all specific to biallelic loss. More importantly, rapamycin does not make any significant effect on the phenotypes in Tsc1 het neurons (Fig 7), so the comments on the effect of Tsc1 heterozygosity should be moderated.
Abstract says "These changes also occur when only one allele of Tsc1 is deleted, making these findings relevant to individuals with TS."
Very few of the changes actually occur in the heterozygous state. This statement should be removed or revised.

We thank the reviewer for pointing this out. In the abstract of the revised manuscript, we have replaced "These changes also occur when only one allele of Tsc1 is deleted, making these findings relevant to individuals with TS." with "Milder intermediate phenotypes also occur…"

2. Page 4: "SST-Cre-lineage cHet and cKO cells in the neocortex had elevated levels of ribosomal subunit S6 phosphorylated at Serines 240 and 244 (Supplementary Figs. 1e-g), indicating increased MTOR activity." There is no quantification in Supplementary Figs. 1e-g to justify this statement.

We have added a graph quantifying these data in Sup. Fig. 1.

3. Page 5: "A similar phenotype of ectopic PV expression in SST+ CINs after Tsc1 deletion was observed in Nkx2.1-Cre; Tsc1 conditional mutants (Fig. S2)." According to the figure, Tsc1 hets do not differ from WT. So, this appears different than the findings in SST-Cre mice.

Please see our response to reviewer 2's point 14.

4. Page 6: "loss of Tsc1 decreased the input resistance (Rin) and produced a corresponding increase in the rheobase (current threshold) of CINs in cHets and cKOs (Figs. 2c, f)."
The only significant differences according to the figures (Fig 1c and 1f) are between WT and cKO, not hets.

We have revised the text in the results sections to correct this.

5. Fig 1q: the authors used 3 mice in each group and report a significant difference between 0% and 2% with this cohort size. The SEM shown on the graph in this panel seems surprisingly small for n of 3. Could you please double-check that n of animals (not n of cells) was used for this graph?

We thank the reviewer for noticing this. We have checked over the data and the SEM reported is correct. Of note, there were several cells counted for this analysis and we have now updated the figure legend to reflect this, by adding the number of cells counted for each genotype, in addition to the "n" of 3, which was the biological replicate number (i.e. number of mice); the large number of cells counted helped assure the percentages measured were very tight, thus leading to small error bars. Moreover, the Chi-squared test used for this type of statistical analysis takes into account the total number of cells assessed, which is why there is a significant difference between the 0.3% in the WT group and the 2.7% in the cHet group.

6. Were the expression of any other ion channels besides Kv3.1 investigated? Please explain the rationale for this specific choice better.

We did not quantify the expression of other ion channels besides Kv3.1 in out study. In comparison to native FS PV+ CINs, SST+ CINs have little to no expression of Kv3.1 channels and also have a higher expression of HCN channels.

In our study, Tsc1 deletion caused a shift in firing properties of SST+ CINs from RS to FS. Previous studies have shown that these properties are specifically coupled to the expression of Kv3.1 channels. Further, Tsc1 deletion did not change HCN channel mediated sag and rebound voltages in these neurons (Supplementary Fig. 7d–f). Based on these data from our physiological analysis and the previous work describing the role of Kv3.1 channel expression in FS physiological properties, we predicted that changes in expression of Kv3.1 channels are the likely ion-channel mechanisms for the shifts in physiology of SST+ CINs.

7. In Figure 3b, were the Kv3.1 + tdTomato expressing cells co-localized with increased PV expression?

Yes, we found a strong correlation between PV and Kv3.1 expression in the tdTomato+ cKO CINs; ~91% PV and Kv3.1 co-labeled within the tdTomato+ cell population. These data now comprise Sup. Fig. 11, panels a-d.

8. Rapamycin dose used in this study (10mg/kg IP every day) is a very high dose based on the PK of rapamycin in the brain. It would be helpful to include a reason why this high dose was chosen.

We chose this high dose of rapamycin to test the acute effects of inhibiting mTOR signaling on SST+ CIN physiology and molecular expression in adult mice. Similar doses have been used in previous mouse studies characterizing the cellular and behavioral effects of increased mTOR signaling (deletion of signaling molecules like Tsc1 and Pten). Zhou et al 2009 paper used 10 mg/Kg rapamycin for 5 days; Sato et al 2012 used 10 mg/Kg rapamycin for 2 days.

Sato A, Kasai S, Kobayashi T, Takamatsu Y, Hino O, Ikeda K, et al. Rapamycin reverses impaired social interaction in mouse models of tuberous sclerosis complex. Nature Communications. 2012. 3:1292.

Zhou J, Blundell J, Ogawa S, Kwon C-H, Zhang W, Sinton C, et al. Pharmacological Inhibition of mTORC1 Suppresses Anatomical, Cellular, and Behavioral Abnormalities in Neural-Specific Pten Knock-Out Mice. J Neurosci. 2009 29(6):1773.

9. Please include a discussion the recent paper by Zhao and Yoshii (PMID: 30683131), which does not find a phenotype in the selective deletion of Tsc1 from either PV or SST cells.

We have included a discussion of this paper in the discussion.

Minor comments:

10. Please use the conventional abbreviation for Tuberous Sclerosis Complex (TSC) for this disorder. TS is not commonly used.

We have changed the abbreviation from TS to TSC in the manuscript.

11. The role of the mTOR complex in transcriptional regulation as it relates to what is known about patterning of the MGE should be briefly discussed. For example, does mTOR dysregulation affect Lhx6 expression (mentioned in the introduction)?

To the best of our knowledge, and not including our work on the Pten gene (Vogt et al., 2015), there is no other literature investigating the role of MTOR signaling in the MGE and on the expression of Lhx6.

12. Line 3 Page 4- missing “we” after “To test this,”

Thank you, we have corrected this.

REVIEWERS' COMMENTS:

Reviewer #1 (Remarks to the Author):

This paper is well revised

Reviewer #2 (Remarks to the Author):

The authors have done an outstanding job addressing all of the reviewer's comments. They demonstrated that simply increasing cell size is insufficient to drive the observed changes in SST+ cells. The inclusions of cell reconstructions and sag/Ih current analysis extend their observations into the amount that these cells adopt PV/FS characteristics. The DREADD experiments reveal an interesting correlation between cell activity and adoption of PV/FS characteristics. This is a very important paper with novel insights regarding mTor function in interneuron development, and has broad implications for how fate decisions are determined in MGE-derived interneurons (and potentially other cell types as well).

Reviewer #3 (Remarks to the Author):

The authors have addressed my comments. The revised manuscript is significantly improved.
Mustafa Sahin
